# Setting targets for antibiotic use in general practice in Europe: A scoping review

**DOI:** 10.1080/13814788.2024.2430507

**Published:** 2024-11-28

**Authors:** Nathaly Garzón-Orjuela, Kevin Roche, Heike Vornhagen, Aoife Moran, Scott Walkin, Walter Cullen, Catherine Blake, Akke Vellinga

**Affiliations:** a CARA Network, School of Public Health, Physiotherapy and Sports Science, University College Dublin, Dublin, Ireland; b School of Public Health, Physiotherapy and Sports Science, University College Dublin, Dublin, Ireland; c School of Psychology, University of Galway, Galway, Ireland; d Insight Centre for Data Analytics, University of Galway, Galway, Ireland; e Irish College of General Practitioners, Dublin, Ireland; f School of Medicine, University College Dublin, Dublin, Ireland

**Keywords:** National action plan, primary health care, general practice, antimicrobial resistance, drug resistance, target, benchmark

## Abstract

**Background:**

National Action Plans (NAPs) aim to address antimicrobial resistance (AMR) understanding and awareness but struggle to translate targets into clinically relevant guidance for general practice.

**Objective:**

To identify and map antibiotic use targets in European general practice and explore if and how these targets are linked to NAPs.

**Methods:**

A systematic search was carried out in MEDLINE (OVID), EMBASE and SCOPUS, with additional manual searches. The research questions were: What are existing targets for antibiotic use in general practice in the 31 European countries? and How are these targets linked to the NAPs on AMR?. The results are presented narratively.

**Results:**

77 reports were included, of which 33 focused on national targets and general practice or linking national and local targets. Reports describe local strategies to achieve targets, such as prescriber feedback, benchmarking systems and financial incentives. However, these reports provide aggregated targets for general practice, such as a percentage reduction of antibiotics prescribed. These targets are set in general, for a specific type of antibiotic, for an amount per number of patients, in defined daily doses or items. None of the reports translate national targets into clinically relevant or practical targets for general practitioners.

**Conclusion:**

Most European countries have an NAP with established targets, but the type and implementation of these targets vary between nations. Translating national targets into daily clinical practice is challenging and often lacks the involvement of prescribers. Aligning national and local targets would enhance coherence and more effectively contribute to improvements in antibiotic use.

## KEY MESSAGES

Translating targets into daily clinical practice is challenging and often lacks the involvement and input of prescribers.

Future research should prioritise collaborations with GPs for setting antibiotic use targets in general practice and development into clinically relevant and practical targets that align with NAP targets.

## Introduction

Inappropriate use of antibiotics drives antimicrobial resistance globally [1–3]. In 2015, the World Health Assembly launched an initiative to develop national action plans (NAPs) on antimicrobial resistance (AMR) [4]. However, the biggest challenge remains the practical implementation of NAPs, into sustainable and actionable targets, which can also be used to measure outcomes and goals [5]. In 2022, the World Health Organisation (WHO) issued guidance on NAP implementation [6], followed by additional guidance in 2023 to enhance the monitoring and evaluation of these plans [7]. This guidance emphasises the importance of identifying indicators, baselines and targets to track stakeholder actions. Furthermore, it highlights the need for the NAPs to consider their intended audience, ensuring that they align with the responsibilities of each country in relation to the Global Action Plan on AMR [6,7].

Focusing on the local implementation and monitoring of actions derived from the NAPs could be a useful approach [5]. Focusing national targets to particular settings or geographical areas would allow targets to be made relevant, appropriate and significant for local stakeholders. A scoping review on performance indicators and targets for antibiotic prescribing in acute hospital settings found a need for more evidence to aid target setting and benchmarking [8].

Relevant factors to set targets include indicator type, pre-existing data, data collection cost and the monitoring of data [9]. However, no specific targets for antibiotic use are set for local settings such as general practice. As the majority of antibiotics are prescribed in general practice [10], it is important to use clinically relevant and practical targets that are measurable and achievable in this setting. Aligning general practice targets with NAPs could more effectively contribute to improvements in overall antibiotic use. Therefore, this scoping review aims to identify and map antibiotic use targets for general practice and explore if and how these targets are linked to NAPs in 31 European countries.

## Methods

The scoping review was conducted using the Joanna Briggs Institute Manual for Scoping Reviews [11] and the framework developed by Arksey and O’Malley [12]. To ensure rigour in reporting, this scoping review adhered to the Preferred Reporting Items for Systematic Reviews and Meta-Analyses extension for Scoping Reviews (PRISMA-ScR) (see the PRISMA-ScR checklist in Supplementary Material 1) [13]. The protocol of this scoping review was registered in Open Science Framework [14].

### Identifying the research questions

Two research questions guided the objective of this scoping review:

Q1. What are existing targets for antibiotic use in general practice, focusing on the 31 European countries of the original European Public Health Alliance overview from 2018 (Table 1)? [5];

**Table 1.** Eligibility criteria.

	Inclusion	Exclusion
Population	All human population groups	–
Concepts	Any sources on targets for antibiotic use. Target is defined as a quantitative measure with a clear commitment to achieving specific results within a predefined time and involving a change in outcomes or processes. This measure could be absolute, proportional, relative to a benchmark, relative to an expected level, relative to cost/value for money, tied to a tolerance threshold or depend on the nature of the data (through modelling or projection and trend analysis) [9].	–
Contexts	General practice Countries: Austria, Belgium, Bulgaria, Croatia, Cyprus, Czech Republic, Denmark, Estonia, Finland, France, Germany, Greece, Hungary, Iceland, Ireland, Italy, Latvia, Lithuania, Luxembourg, Malta, Netherlands, Norway, Poland, Portugal, Romania, Slovakia, Slovenia, Spain, Sweden, Switzerland, United Kingdom	–
Types of evidence sources	Research studies (originals and reviews) National Action Plans Dissertations/theses (via https://opengrey.eu/)	Conference abstracts Editorials, commentaries and opinion papers
Languages	No language restrictions	–

Q2. How are the existing targets (Q1) linked to the NAPs on AMR?

### Identifying relevant articles

This scoping review search was conducted in the following electronic research databases: MEDLINE (OVID), EMBASE and SCOPUS, utilising a comprehensive search strategy designed based on relevant keywords and their variants for ‘antibiotic’, ‘target’ and ‘primary care or general practice’. These keywords were selected from a previous review of relevant papers on the topic (see Supplementary Material 2) [9,15–17].

A manual search of the relevant websites of professional bodies and public health organisations (see Supplementary Material 3) was carried out. Furthermore, reference lists of included studies were examined for additional papers to be considered for inclusion. A search of grey literature was conducted by running keyword searches in OpenGrey, including technical or research reports, doctoral dissertations and some official publications. The existing NAPs on AMR in the 31 European countries (Q2) were searched to identify specific targets for general practice through a manual search of the WHO library or national databases in each country. If a NAP was outdated and did not report a target, the tracking AMR Country Self-Assessment Survey (TrACSS) report was checked for more information. The cut-off point for inclusion was 6 May 2024.

### Study or report selection

After the deletion of duplicates, the electronic database search results were screened in two stages using the Covidence platform [18]. Title and abstracts were independently screened by two reviewers (N.G.O. and K.R.), assessing each report according to specified eligibility criteria (Table 1). The second stage was a full-text screening, where each included report from the first phase was screened by the two reviewers independently (N.G.O. and K.R.). In case of a disagreement in any of the phases, both reviewers discussed disagreements and if the conflict is not resolved, a third author (A.V.) was consulted for resolution. Reports that appear relevant from the second screening process but did not meet the inclusion criteria were listed in the table of excluded reports with reasons for their exclusion and reported in the PRISMA – ScR flow diagram.

The records from other resources (manual search, grey literature and NAPs) were assessed for eligibility by one researcher (N.G.O.) and checked by a second researcher (A.M.) (Table 1).

### Charting the data

The data extraction form included an ID; the actual general practice target and national target; population and setting; methodologies used to develop targets (if reported); if targets are specific, measurable, accurate, realistic and time-bound (SMART) [9]; how targets are evaluated and monitored; and if targets are disease-specific. One researcher (N.G.O.) extracted the data.

### Collating, summarising and reporting results

Extracted data was recorded in MS Excel. The results were presented narratively in response to the scoping review research questions. This scoping review included understanding the nature, completeness and com­plexity of the information regarding antibiotic use. Therefore, a quality assessment was outside the scope of this review. Even though no one standard approach can be defined for target setting, policymakers and researchers should set targets based on several factors. Therefore, when the target setting was evident in the included reports, a checklist was used to check factors to consider when setting targets [9]. R version 4.3.3 was used to generate a European map illustrating the results [19]. The ‘Online Doc Translator’ was used to translate any reports that were not published in English or Spanish [20].

## Results

**Figure 1.**
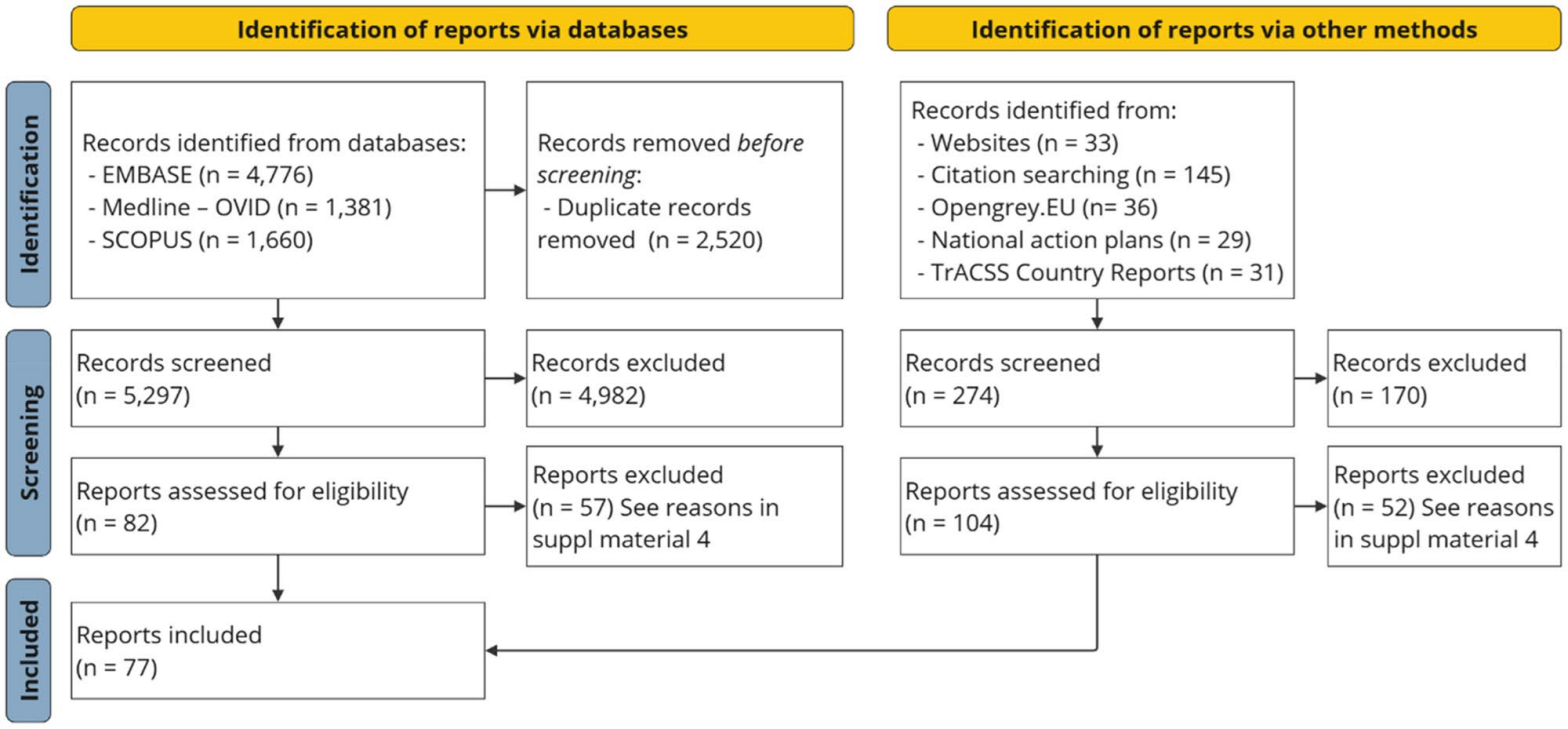
PRISMA – ScR flow diagram [13].

After removing duplicates, a total of 5,571 potentially relevant reports were identified. After assessing against the eligibility criteria, 77 reports were included in the synthesis of evidence (Figure 1). The list of excluded reports and the reasons for their exclusion are shown in Supplementary Material 4. In general, 71% of exclusions were due to studies not targeting antibiotic use.

**Figure 2.**
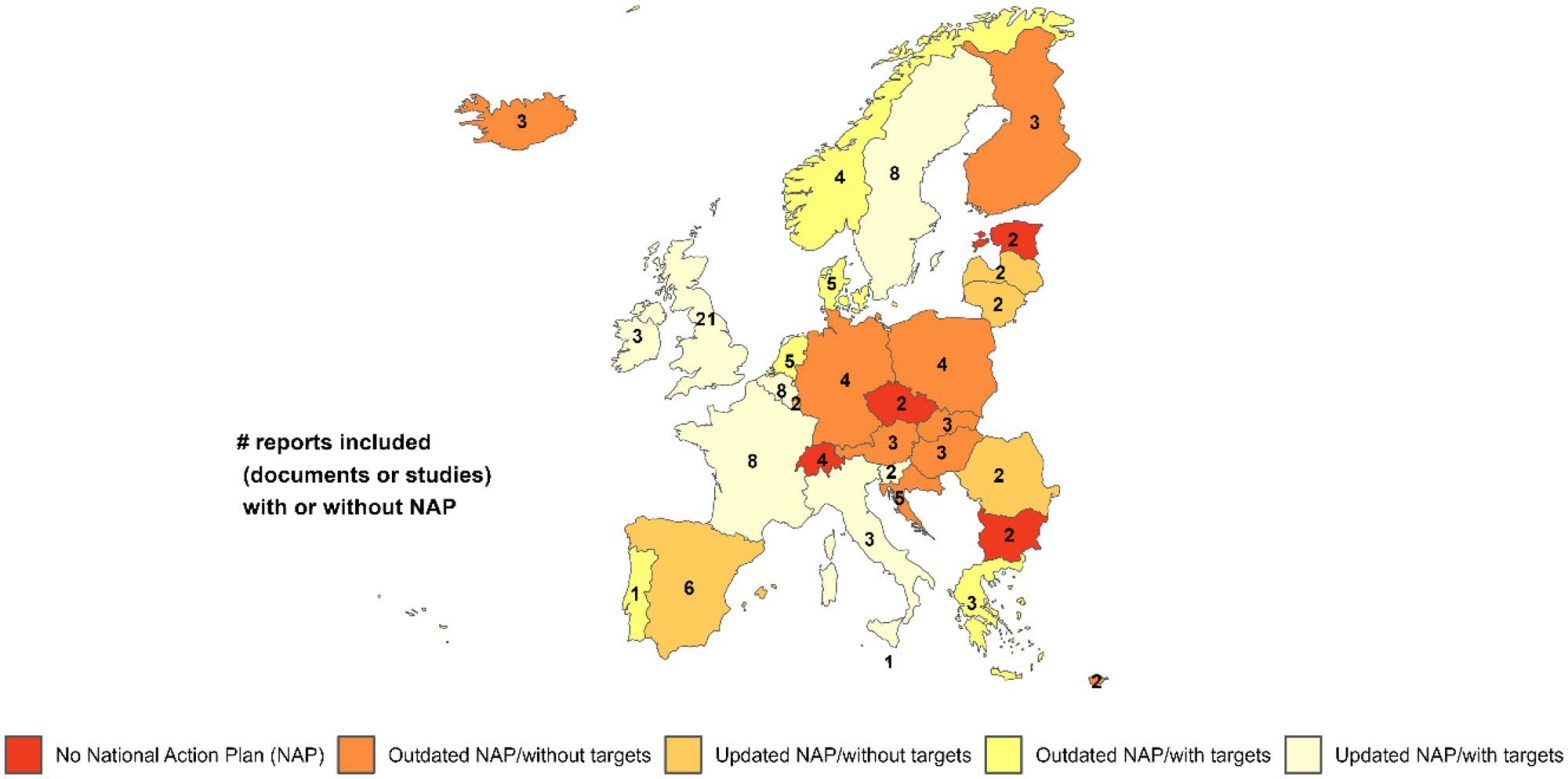
Number of reports and national action plans with or without targets by country.

Of the 77 reports, 36 were documents or studies (that included multiple countries in some cases) and 41 were NAPs and TrACSS reports that included national, general practice (local), or strategies to link national targets to local areas (Figure 2). Latvia, Lithuania, Luxembourg, Malta, Romania and Spain had updated NAPs but no antibiotic use targets. Austria, Croatia, Cyprus, Finland, Germany, Hungary, Iceland, Poland and Slovakia had outdated NAPs without targets. Bulgaria, Czech Republic, Estonia and Switzerland’s NAPs were not found. The TrACSS report provided more details on AMR NAPs for these countries (see Supplementary Material 5 and Figure 2).

Table 2 shows national, regional and local targets relevant to general practice. France, Ireland, Sweden and the UK reported national and local targets (Table 2). Belgium, Denmark, Greece, Italy, the Netherlands, Norway, Portugal and Slovenia reported only national or regional targets (see Supplementary Material 6).

**Table 2.** National, regional and local targets.

Country	National/regional targets	General practice target
France	Community care *2022 to 2025* - Reduce the total consumption of all antibiotics by at least 20 DDD per 1,000 inhabitants per day by 2025 [47] - Maximum 20% of antibiotic treatments per 100 patients (attending physician) aged 16 to 65 years old and with no long-term illness [47] - Reduce to 650 or less the number of antibiotic prescriptions dispensed in community care (per 1,000 inhabitants and per year) [47] - Maximum 20% of the consumption of all critical antibiotics for systemic use in community care, in DDD per 1,000 inhabitants per day, dispensed in community pharmacies by 2025 [47] Primary care *2011 to 2017* - Reduce to 14 treatments per 100 patients the annual antibiotic prescription rate in patients aged 16–65 years without a chronic disease [30,44] - Maximum 27% of patients treated with ‘critical antibiotics’ (amoxicillin-clavulanic acid, third- and fourth generation cephalosporins, fluoroquinolones) [30,44] - Maximum 3% of children treated with third generation cephalosporins in one year, out of children receiving antibiotics (any child aged < 4 years) [30,44] - Maximum 2% of children treated with third generation cephalosporins in one year, out of children receiving antibiotics (any child aged ≥4 years) [30,44]	*2011 to 2017* - Reduce to 14 treatments per 100 patients the annual antibiotic prescription rate in patients aged 16–65 years without a chronic disease [30,44] - Maximum 27% of patients treated with ‘critical antibiotics’ (amoxicillin-clavulanic acid, third- and fourth generation cephalosporins, fluoroquinolones) [30,44] - Maximum 3% of children treated with third generation cephalosporins in one year, out of children receiving antibiotics (any child aged < 4 years) [30,44] - Maximum 2% of children treated with third generation cephalosporins in one year, out of children receiving antibiotics (any child aged ≥4 years) [30,44]
Ireland	*2022 to 2025* Community care - Reduce antibiotic prescribing by 2% each year to achieve a target of 20.5 DDD by 2025 [48]	*2022 to 2025* - Reduce ‘red’ antibiotics by 8% (to 26%) by 2025 [48] - Reduce the total number of antibiotic prescriptions by 8% for patients receiving free healthcare by 2025 [48]
Sweden	Primary care/Outpatient care/Ambulatory care - Maximum 250 antibiotic prescriptions issued per 1,000 inhabitants per year [44,46,49–52] - Minimum of 80% of all antibiotics used to treat respiratory tract infections in children aged 0–6 years should be penicillin V (J01CE02) [44,49–52] - Maximum 10% of all antibiotics used to treat urinary tract infections in women aged 18–79 years should be fluoroquinolones [44,49–52] - Maximum 10% of patients with acute bronchitis should receive antibiotic treatment* [44,50] - Maximum 80% of women and more than 50% of men with afebrile urinary tract infection should receive first-line treatment* [44] - Maximum 90% of patients treated against pharyngotonsillitis should receive penicillin V* [44] *Targets suggested by the Strama Programme Council operational plan	- Maximum 20% of all acute bronchitis should be treated with antibiotics [51] - Over 70% of all patients treated with antibiotics for pneumonia should receive penicillin V [51] - Maximum 3% of women over 18 years of age who are treated with quinolones when diagnosed with cystitis. The same applies to the proportion of women with cystitis who are treated with cephalosporins [51]
United Kingdom (UK)	*2013 to 2018* - Reduce inappropriate antibiotic prescriptions by 50% by 2020 [28,44] 2019 to 2024 - Reduce the total number of antibiotic-resistant infections by 10% from the 2018 baseline by 2025 [43,53] - Reduce total antimicrobial consumption in humans by 15% by 2024, from a 2014 baseline [39,43,53–57] - Reduce antibiotic use in the community by 25% by 2024 [43,54] In order to implement the national strategy, England and Scotland developed its own objectives [44] Ambulatory care/primary care - England - Maximum 1.161 items per STAR-PU of the 2013-2014 baseline mean performance value for England (a 1% reduction) [30,39,44,58]. Reduced to 0.965 in 2019-2020 [56,59]. In 2022-2023, it was further reduced to 0.871 items per STAR-PU [43] - Maximum 10% of broad-spectrum antibiotic prescribing (co-amoxiclav, cephalosporin class and fluoroquinolone) [30,39,43,56,59] - Reduce inappropriate antibiotic prescribing for urinary tract infections, the target for 2017–18 (based on June 2015–May 2016 baseline data) includes a minimum 10% reduction in the trimethoprim/nitrofurantoin prescribing ratio and a 10% decrease in trimethoprim prescriptions for patients aged ≥ 70 years due to higher trimethoprim non-susceptibility rates in this age group [30,44] Ambulatory care / Primary care - Scotland - Practices must achieve an equivalent or lower prescribing rate to the Scottish 25th percentile or reduce their prescribing rate by at least one-fifth of the national interquartile range [44] - Seasonal variation in quinolone use (summer months (April–September) vs. winter months (October–March)) is < 5% [60,61]	England - Maximum 10% of broad-spectrum antibiotic prescribing (co-amoxiclav, cephalosporin class and fluoroquinolone) [30,55,56] - Maximum 1.161 items per STAR-PU of the 2013-2014 baseline mean performance value for England (a 1% reduction) [30,58]. Reduced to 0.965 in 2019–2020 [56,58,59] Scotland - Seasonal variation in quinolone use (summer months (April–September) vs. winter months (October–March)) is < 5% [60,61]

STAR-PU: Specific Therapeutic group Age-sex Related Prescribing Unit; DDD: defined daily doses.

General practice targets in France are in line with primary care targets but differ from national community care targets (Table 2). Concerning the SMART criteria, the local targets were specific and trackable. However, the precision of measurement, achievability, and time-bound nature of these targets were uncertain. Additionally, the information provided did not adequately complete the target-setting checklist (see Supplementary Material 7). Moreover, visual data analytic tools, pay-for-performance targets, the development of indirect proxy indicators and feedback to prescribers are strategies that have been implemented in general practice to help align with the national target (Table 3). Four academic research studies were identified [21–24], with three of them suggesting optimal and acceptable targets for the appropriateness of antibiotic use. These targets were established based on routine data, proxy indicators, and expert opinion [21–23]. Arias P et al. utilised a cut-off value for three indicators pertaining to the overall antibiotic prescriptions per visit and the frequency of prescribed antibiotic types. This approach aimed to identify instances of excessive antibiotic use, particularly those with a high AMR impact [24]. It is noteworthy, however, that while these targets were research-oriented, they were not directly associated with the NAP initiative [21–24].

**Table 3.** Strategies or initiatives to help linking the national target to the local area.

Country	Organisation	Tool or system	Financial incentives	Interventions
Belgium	–	–	–	- National cost-effective web-based communication skills training to support general practitioners to safely reduce antibiotic prescribing in patients presenting to primary care with acute respiratory tract infections [62]
Denmark	–	–	–	- The ‘wait-and-see’ prescription is similar to a regular prescription, but the physician advises the patient not to fill it immediately (depending on symptoms) [63]
France	–	- Indicator dashboard with targets to guide the national/regional/local strategy for infection prevention and control and antibiotic stewardship and to improve the national and regional sharing of available data and indicators to promote their use for taking action [47]	- Pay for performance target for general practices [44]	- Developing indirect proxy indicators of the appropriateness of antibiotic prescriptions to be compiled routinely in the three care sectors and accompanied by an aggregate presentation of the results and feedback to prescribers [47,64] - HAPPY PATIENT project which is focus on decreasing inappropriate antimicrobial use for common community-acquired infections [64]
Italy	–	–	–	- Audit and Feedback for antibiotic prescribing is a required institutional activity in each Local Health Authority [64]
Ireland	The Antimicrobial Resistance Infection Control core team reviews and monitors the implementation of projects and actions [48]	–	–	- Guidance on preferred antibiotics in community (Red/Green GP antibiotic prescribing reports) [48] - Prescriber feedback: inform behavioural change and support extension of best practice projects, such as GP preferred antibiotics prescribing [48]
Netherlands	–	–	–	- A national voluntary programme for general practitioners to provide data from their charts and get structured feedback and discussion in small groups [64]
Norway	–	–	–	- Audit and Feedback for GPs and nursing homes (more appropriate antibiotic use in municipalities and nursing homes) [64]
Spain	–	–	- Pay-for-performance, meaning that general practitioners get a financial bonus if the target is reached [30]	- HAPPY PATIENT project [64]
Sweden	Strama, an interdisciplinary organisation overseeing antibiotic use, provides peer-to-peer feedback to prescribers on their adherence to treatment recommendations and local targets. They conduct stewardship interventions based on local data [49–51,64]	Primary Care Quality is a user-friendly quality system with diagnosis-linked indicators and feedback for continuous improvement. It shows benchmarks on local, regional and national levels, with targets monitored via the Primary Care Quality national register. Its primary purpose is to facilitate easy follow-up and local quality improvement without extra administrative work for primary care professionals [44,50,51,64]	County councils can earn performance-based reimbursement from the state if they meet three conditions: [46] 1. Supporting local multiprofessional groups with written mandates and funding 2. Increasing prescriber adherence to treatment recommendations 3. Providing individual antibiotic prescribing feedback to at least half of the healthcare centre general practitioners and benchmarking prescribing at all centres.	–
United Kingdom (UK)	The National Health Service (NHS) England, in collaboration with the NHS Business Services Authority, reports on integrated care boards (ICB) performance for antimicrobial resistance (AMR) indicators to monitor antibiotic prescribing in primary care and track ICB progress towards NHS Oversight Framework targets [43]	England - Free-access monthly practice-level prescribing databases like ‘Fingertips’ and ‘ePact2’ enable prescribers to benchmark their practice against local, regional and national levels [39,53,64–66] - PrescQIPP: Antimicrobial Stewardship (AMS) Visual Analytics [39,55] - Lowering AntiMicrobial prescribing (LAMP) is a regional UK project, which utilises medical record data [64]	England Financial incentives linked to specific objectives and targets (local financial incentive schemes) [30,39,44,56,67,68]	Scotland National targets prompted local primary care interventions within Health Boards, primarily aiming to reduce use of the ‘4C’ (co-amoxiclav, ciprofloxacin, cephalosporins and clindamycin). They also encourage reviewing variation within teams and comparing with similar practices, implementing changes to meet agreed targets [43,60] - Feedback provided to Scottish general practices compares their prescribing data to benchmarks based on the 25th percentile, representing the antibiotic prescribing rate achieved or surpassed by the lowest-prescribing quarter of practices within their local National Health Service board and across Scotland as a whole [65] England - Routine Audit and Feedback for antibiotic prescribing for primary care [39,64,67,68] - ‘Treat Antibiotics Responsibly, Guidance, Education and Tools’ (TARGET) audits for prescriber self-assessment [39,43,64,67,68] - Quality Premium (QP) improvement measures [39,67,68] - Practitioners encouraged to become antibiotic guardians [67] - AMS/AMR education/learning [39,67] - Completion of a self-assessment checklist [67] - Social norm feedback about broad spectrum prescribing [39,59,68] - Local antibiotic use guidance [39]

In Ireland, the local targets are consistent with the national community care targets and also include a target that links to the Irish national guidelines. These guidelines categorise antibiotics into ‘green’ (preferred - antibiotics that are more effective, have fewer side effects, and are less likely to lead to resistant infections) and ‘red’ (non-preferred - antibiotics that should not be used in primary care unless absolutely necessary) [25,26]. However, the Irish reports did not provide extensive information regarding the SMART criteria and the target-setting process (see Supplementary Material 7).

The general practice targets in Sweden differed from national targets (Table 2). In the SMART criteria, Swedish targets lacked specified timing (see Supplementary Material 7). Additionally, the target-setting checklist lacked clarity regarding reference values or baselines, as well as current and historical data trends to define targets (see Supplementary Material 7). Nevertheless, Swedish reports indicate the presence of local strategies or initiatives aimed at target achievement. These include feedback to prescribers, a system facilitating indicators, feedback benchmarks at local, regional and national levels (primary care quality) and financial incentives (Table 3).

The local targets in the UK, England and Scotland, is consistent with the primary care targets but differed from national targets (Table 2). In England, targets were specific, measurable, accurate, realistic and time-bound (see Supplementary Material 7). The targets provided information about baseline, trends, local initiatives and how these targets were adjusted over time. However, they lacked clarity regarding the cost of data collection and whether achievement of the target can be considered good value in comparison with other approaches or interventions (see Supplementary Material 7). Two academic research studies used a variety of benchmarks, such as antibiotic prescribing appropriateness and the ideal proportion of consultations resulting in systemic antibiotic prescriptions, but these targets were research-focused and not linked to the NAP targets [27,28]. Feedback provided to GPs in the England highlight local strategies or initiatives, including visual data analytic tools that incorporate benchmarks against local, regional and national standards, routine audit and feedback for prescribers and financial incentives (Table 3).

Two reports from Spain referenced local targets aimed at reducing the total and second and third-line antibiotic prescriptions (macrolides, cephalosporins, fluoroquinolones) [29,30]. However, these targets were not measurable, accurate, realistic or time-bound [29,30].

On the other hand, seven academic studies used the acceptable range values proposed by the European Surveillance of Antimicrobial Consumption project (ESAC) [31] to monitor existing data or make comparisons between countries [32–38]. However, the methodology behind these acceptable ranges was unclear and focused on outpatient settings. The authors of the ESAC project suggested that using these ranges as a true benchmark should be approached with caution. Various contextual factors in primary care settings, such as local guidelines and different thresholds for consulting a GP, may influence this acceptable range [31]. In SMART criteria, the acceptable ranges were specific and could be monitored. However, accurate measurement, achievable and timebound were unclear. Furthermore, it did not provide too much information to fill in the target-setting checklist.

## Discussion

Available information on how existing antibiotic use targets in general practice are linked to NAP initiatives are limited. This finding highlights a gap between prescribing targets and how they are applied in practice. Reports provide aggregated targets, such as reduction of number or overall percentage of antibiotic prescribing, in general or for a specific type of antibiotic, or an amount per number of patients, in defined daily doses (DDD) or items. However, the question remains as to how this translates into daily clinical practice considering outcomes such as a 10 or 20% reduction in antibiotic prescribing or decrease to 10 or 20 antibiotic treatments per 100 patients annually. The lack of standardised national and international targets, the diversity in prescribing measures and data sources and the complexity of implementing stewardship policies in prescribing patterns contribute to the existing gap between antibiotic prescribing targets and their practical application in daily clinical practice [39].

The reports included did not explicitly translate targets into clinical practice guidance for GPs, nor did they provide clear instructions on how GPs can participate in target development. For antibiotic use target setting, it is crucial that prescribers (GPs) are involved in the process to ensure that their performance is accounted for. For national antibiotic use targets to translate into meaningful and achievable outcomes in clinical practice, it is essential to actively involve GPs and staff in the target-setting process, provide them with relevant data and information, and support them through tailored action planning and resources [9,40]. An additional challenge for general practice is how differences in practice population (age, indication and other demographic factors etc.) result in differences in prescribing and therefore targets [41,42]. For instance, older practice populations may require antibiotics more often [42]. This means population specific or adjustable targets are important for GPs to make these targets clinically relevant.

The validity of a target should be guided by various critical factors that shape long-term outcomes and assess the target’s appropriateness. Technical considerations are pivotal among these factors. When aiming for a 10 or 20% reduction, understanding the practical implications of such changes is crucial. There is a potential risk where even a minor adjustment could lead to a significant percentage impact, potentially distorting the target’s interpretation [9]. England stood out as the sole country that reported adjusted targets based on primary care antibiotic prescriptions over time. Despite England meeting the target of ‘at or less than 10%’ for primary care prescribing of broad-spectrum antibiotics, the total primary care antibiotic prescribing for England surpassed the national target [43]. The data reveal an increase to 0.984 items per Specific Therapeutic group Age-sex Related Prescribing Unit (STAR-PU), which is 15% below the 2013-2014 baseline but still reflects a notable rise of 5 million primary care antibiotic prescriptions in the 12 months to 31 March 2023 compared to the previous year [43].

This highlights a significant distinction between national-level targets, plans and overviews, and actual GP prescribing practices on the ground. While national-level data may indicate an overall change in specific prescribing targets, the practical implications at the local level can vary. To effectively assess antibiotic prescribing targets, it is essential to analyse both national statistics and detailed local prescribing data. This granular analysis can determine whether national strategies result in meaningful changes in clinical practice. A comprehensive examination of both macro-level and micro-level data provides a nuanced understanding of antibiotic stewardship initiatives and their impact [39,44]. This integrated analysis is vital for evaluating the validity and effectiveness of the established targets. Additionally, to improve national targets, aligning specific general practice targets with NAP, could support an integrated and potentially more successful approach.

Fourteen academic research studies have used benchmarks, cut-offs, optimal or acceptable values to compare antibiotic usage across different countries or to establish goals for ideal or appropriate antibiotic consumption [21–24,27–29,32–38]. Nevertheless, none of these studies have explicitly connected these targets or values to the NAP targets. Furthermore, none of the reports provide detailed information regarding the cost of data collection and how it compares to achievements in reaching the target in terms of value for money. A recent systematic review of economic evaluation studies focusing on interventions impacting AMR revealed a significant evidence gap in the economics of AMR [45]. This emphasises the need for context-specific cost-effectiveness analyses to assess the value for money of various interventions to reduce antibiotic prescribing [45].

A key lesson from this scoping review is the importance of clinically relevant targets based on guidelines, supported by robust evidence and data, and developed through a bottom-up approach that closely involves prescribers at the local level. Targets should be credible and acceptable for stakeholders, grounded in epidemiological studies, diagnosis-related prescription data, representative patient populations, and national guidelines. Regular re-evaluation and adjustment are crucial as primary care data, clinical practices, and epidemiological factors evolve. Ultimately, a collaborative, data-driven, and adaptable approach is key to implementing an effective national antibiotic use target that can drive meaningful and sustainable improvements in antimicrobial stewardship [9,27,46].

Furthermore, it is notable that there is a lack of enabling infrastructure and system tools to help link the national target to the local area. Only Sweden, the UK, and France mentioned the development of such infrastructure, which include benchmarks on local, regional, and national levels. However, future infrastructure should be developed to help achieve this goal of linking the national target to the local area, including the involvement of GPs in target-setting [69].

This scoping review employed a comprehensive search strategy across databases, websites, and reports without language restrictions. However, it is essential to acknowledge the potential for researcher bias, as data extraction was performed by only one researcher (N.G.O.). Scoping reviews often allow for greater flexibility in their methodology, as they aim to capture a broad range of evidence. Also the potential for publication bias, as unpublished grey literature or internal reports from included countries may have been excluded, potentially leading to an incomplete representation of available evidence.

## Conclusion

Most European countries establish NAPs with targets, yet the nature and implementation of these targets vary considerably between nations, contributing to disparities at local, regional and national levels. The diversity in prescribing measures, the absence of standardised national and international targets and the complexity of implementing stewardship policies exacerbate this variance. The active involvement of GPs in target setting is crucial for ensuring their commitment and responsibility to achieving these goals. To facilitate successful implementation, there is a clear need for clinically practical targets for GPs, with their active participation in target development, and tailored support through action planning and resource allocation. Future research should prioritise collaborations with GPs to establish an international consensus approach for setting antibiotic use targets in general practice and development into clinically relevant and practical targets. Additionally, it is important to work with GPs to create strategies that address the challenges of high workloads and ensure the implementation of these targets is feasible in everyday practice. By identifying best practices in target development, future research can offer insights to policymakers on effectively addressing challenges in healthcare services.

## Supplementary Material

Supplemental Material
