# Supplementary material for: Setting targets for antibiotic use in general practice in Europe: A scoping review
Source: Eur J Gen Pract. 2024 Nov 28;30(1):2430507. doi: 10.1080/13814788.2024.2430507 (PMC11610282; doi:10.1080/13814788.2024.2430507)
Supplement: Supplemental Material [file IGEN_A_2430507_SM3297.zip › ejgp-2024-0174-File004.docx]

Supplementary Material 2. Evidence search report in electronic databases

| **Electronic search report No. 1** | |
| --- | --- |
| **Electronic database** | MEDLINE(R) ALL |
| **Platform** | OVID |
| **Date of search** | 06-05-2024 |
| **Range of date** | 1946 to May 06, 2024 |
| **Restriction of language** | None |
| **Other limits** | None |
| **Search strategy (results)** | 1. exp Anti-Infective Agents/ (1861641) 2. exp Antibiotic Prophylaxis/ or exp Anti-Bacterial Agents/ (844533) 3. antiinfective agent*.ab,ti,kw. (113) 4. Antibiotic*.ab,ti,kw. (446917) 5. Antiinfective*.ab,ti,kw. (622) 6. Anti infective*.ab,ti,kw. (7390) 7. Antimicrobial*.ab,ti,kw. (242610) 8. Anti microbial*.ab,ti,kw. (6351) 9. Antibacterial*.ab,ti,kw. (116602) 10. Anti bacterial*.ab,ti,kw. (6264) 11. antibiotic* use.ab,ti,kw. (20427) 12. antimicrobial* use.ab,ti,kw. (4420) 13. antibiotic agent*.ab,ti,kw. (2063) 14. antibiotic therapy.ab,ti,kw. (40497) 15. 1 or 2 or 3 or 4 or 5 or 6 or 7 or 8 or 9 or 10 or 11 or 12 or 13 or 14 (2164787) 16. threshold*.ab,ti,kw. (340246) 17. (improvement and target*).ab,ti,kw. (60535) 18. (set and target*).ab,ti,kw. (76064) 19. target*.ab,ti,kw. (2151773) 20. (target* adj2 optimal).ab,ti,kw. (3171) 21. (target* adj2 acceptable).ab,ti,kw. (306) 22. (Range adj2 optimal).ab,ti,kw. (3271) 23. (Range adj2 acceptable).ab,ti,kw. (4018) 24. (target* adj2 value*).ab,ti,kw. (5351) 25. (target* adj2 performance).ab,ti,kw. (2407) 26. cut off value*.ab,ti,kw. (33658) 27. (target* and compliance).ab,ti,kw. (10178) 28. appropriateness score.ab,ti,kw. (46) 29. ((range adj2 acceptable) and value*).ab,ti,kw. (1241) 30. benchmarking.ab,ti,kw. (13275) 31. outcome and process assessment.ab,ti,kw. (229) 32. exp Goals/ (20524) 33. Action plan.ab,ti,kw. (6213) 34. 16 or 17 or 18 or 19 or 20 or 21 or 22 or 23 or 24 or 25 or 26 or 27 or 28 or 29 or 30 or 31 or 32 or 33 (2539972) 35. exp Primary Health Care/ (197101) 36. exp General Practice/ or exp Family Practice/ (79281) 37. exp Physicians, Family/ or exp General Practitioners/ (28553) 38. Primary Health Care.ab,ti,kw. (35520) 39. Primary Care.ab,ti,kw. (152058) 40. Primary Healthcare.ab,ti,kw. (10740) 41. General Practitioner*.ab,ti,kw. (59547) 42. General Practice*.ab,ti,kw. (48734) 43. primary medical care.ab,ti,kw. (986) 44. (European adj2 countr*).ab,ti,kw. (34348) 45. ((Belgium or Greece or Lithuania or Portugal or Bulgaria or Spain or Luxembourg or Romania or Czech Republic or France or Hungary or Slovenia or Denmark or Croatia or Malta or Slovakia or Germany or Italy or Netherlands or Finland or Estonia or Cyprus or Austria or Sweden or Ireland or Latvia or Poland or United Kingdom or Iceland or Norway or Switzerland or Northern Ireland or England) and (primary care or general practice)).ab,ti,kw. (23366) 46. 35 or 36 or 37 or 38 or 39 or 40 or 41 or 42 or 43 or 44 or 45 (453570) 47. 15 and 34 and 46 (1381) |
| **Number of references found** | 1381 |
| **Electronic search report No. 2** | |
| **Electronic database** | Embase |
| **Platform** | Elsevier |
| **Date of search** | 06-05-2024 |
| **Range of date** | None |
| **Restriction of language** | None |
| **Other limits** | None |
| **Search strategy (results)** | 1. antiinfective agent'/exp (5076916) 2. anti-infective agent*':ab,ti,kw (2079) 3. antibiotic prophylaxis'/exp (41077) 4. anti-bacterial agent*':ab,ti,kw (1807) 5. antibiotic agent'/exp (1998621) 6. antibiotic*':ab,ti,kw (603235) 7. antiinfective*:ab,ti,kw (10733) 8. anti infective*':ab,ti,kw (9586) 9. antimicrobial*:ab,ti,kw (318191) 10. anti microbial*':ab,ti,kw (10318) 11. antibiotic therapy'/exp (161474) 12. antibacterial*:ab,ti,kw (155693) 13. anti bacterial*':ab,ti,kw (9829) 14. antibiotic* use':ab,ti,kw (24548) 15. antimicrobial* use':ab,ti,kw (5799) 16. #1 OR #2 OR #3 OR #4 OR #5 OR #6 OR #7 OR #8 OR #9 OR #10 OR #11 OR #12 OR #13 OR #14 OR #15 (5296001) 17. threshold*:ab,ti,kw (451888) 18. improvement:ab,ti,kw AND target*:ab,ti,kw (101427) 19. set:ab,ti,kw AND target*:ab,ti,kw (114521) 20. target*:ab,ti,kw (2885408) 21. (target* NEAR/2 optimal):ab,ti,kw (4859) 22. (target* NEAR/2 acceptable):ab,ti,kw (503) 23. (range NEAR/2 acceptable):ab,ti,kw (5546) 24. (range NEAR/2 optimal):ab,ti,kw (4076) 25. (target* NEAR/2 performance):ab,ti,kw (2909) 26. cut off value*':ab,ti,kw (58375) 27. target*:ab,ti,kw AND compliance:ab,ti,kw (19037) 28. appropriateness score':ab,ti,kw (71) 29. ((range NEAR/2 acceptable):ab,ti,kw) AND value*:ab,ti,kw (1734) 30. benchmarking'/exp (20246) 31. outcome and process assessment':ab,ti,kw (714) 32. goal*:ab,ti,kw (644372) 33. action plan':ab,ti,kw (10269) 34. #17 OR #18 OR #19 OR #20 OR #21 OR #22 OR #23 OR #24 OR #25 OR #26 OR #27 OR #28 OR #29 OR #30 OR #31 OR #32 OR #33 (3928694) 35. primary health care'/exp (216811) 36. general practice'/exp (93712) 37. family practice':ab,ti,kw (10539) 38. physicians family':ab,ti,kw (471) 39. general practitioner'/exp (123969) 40. primary health care'/exp (216811) 41. primary medical care'/exp (138056) 42. primary care':ab,ti,kw (208830) 43. primary healthcare':ab,ti,kw (13523) 44. general practitioner*':ab,ti,kw (81246) 45. general practice'/exp (93712) 46. (european NEAR/2 countr*):ab,ti,kw (48162) 47. (belgium:ab,ti,kw OR greece:ab,ti,kw OR lithuania:ab,ti,kw OR portugal:ab,ti,kw OR bulgaria:ab,ti,kw OR spain:ab,ti,kw OR luxembourg:ab,ti,kw OR romania:ab,ti,kw OR 'czech republic':ab,ti,kw OR france:ab,ti,kw OR hungary:ab,ti,kw OR slovenia:ab,ti,kw OR denmark:ab,ti,kw OR croatia:ab,ti,kw OR malta:ab,ti,kw OR slovakia:ab,ti,kw OR germany:ab,ti,kw OR italy:ab,ti,kw OR netherlands:ab,ti,kw OR finland:ab,ti,kw OR estonia:ab,ti,kw OR cyprus:ab,ti,kw OR austria:ab,ti,kw OR sweden:ab,ti,kw OR ireland:ab,ti,kw OR latvia:ab,ti,kw OR poland:ab,ti,kw OR 'united kingdom':ab,ti,kw OR iceland:ab,ti,kw OR norway:ab,ti,kw OR switzerland:ab,ti,kw OR 'northern ireland':ab,ti,kw OR england:ab,ti,kw) AND ('primary care':ab,ti,kw OR 'general practice':ab,ti,kw) (32531) 48. #35 OR #36 OR #37 OR #38 OR #39 OR #40 OR #41 OR #42 OR #43 OR #44 OR #45 OR #46 OR #47 (507981) 49. #16 AND #34 AND #48 (4776) |
| **Number of references found** | 4776 |
| **Electronic search report No. 3** | |
| **Electronic database** | SCOPUS |
| **Platform** | SCOPUS |
| **Date of search** | 06-05-2024 |
| **Range of date** | None |
| **Restriction of language** | None |
| **Other limits** | None |
| **Search strategy (results)** | 1. TITLE-ABS-KEY ( "anti-infective agent*" OR "antibiotic prophylaxis" OR "anti-bacterial agent*" OR "antiinfective agent*" OR "antibiotic*" OR "antiinfective*" OR "anti infective*" OR "antimicrobial" OR "anti microbial*" OR "antibacterial*" OR "anti bacterial*" OR "antibiotic* use" OR "antimicrobial* use" OR "antibiotic agent*" OR "antibiotic therapy" ) (1536753) 2. TITLE-ABS-KEY ( "threshold*" OR ( "improvement" AND "target*" ) OR ( "set" AND "target*" ) OR "target*" OR ( "target*" W/2 "optimal" ) OR ( "target*" W/2 "acceptable" ) OR ( "range" W/2 "optimal" ) OR ( "range" W/2 "acceptable" ) OR ( "target*" W/2 "value*" ) OR ( "target*" W/2 "performance" ) OR "cut off value*" OR ( "target*" AND "compliance" ) OR "appropriateness score" OR ( ( "range" W/2 "acceptable" ) AND "value*" ) OR "benchmarking" OR "outcome and process assessment" OR "goals" OR "action plan" ) (6402325) 3. TITLE-ABS-KEY ( "primary health care" OR "general practice" OR "family practice" OR "family physicians" OR "primary care" OR "primary healthcare" OR "general practitioner*" OR "primary medical care" OR ( "european" W/2 "countr*" ) OR ( ( "belgium" OR "greece" OR "lithuania" OR "portugal" OR "bulgaria" OR "spain" OR "luxembourg" OR "romania" OR "czech republic" OR "france" OR "hungary" OR "slovenia" OR "denmark" OR "croatia" OR "malta" OR "slovakia" OR "germany" OR "italy" OR "netherlands" OR "finland" OR "estonia" OR "cyprus" OR "austria" OR "sweden" OR "ireland" OR "latvia" OR "poland" OR "united kingdom" OR "iceland" OR "norway" OR "switzerland" OR "northern ireland" OR "england" ) AND ( "primary care" OR "general practice" ) ) (565486) 4. 1 AND 2 AND 3 (1660) |
| **Number of references found** | 1660 |
| **Search report No. 4** | |
| **Electronic** | OpenGrey MySQL |
| **Platform** | DANS EASY Archive  <https://easy.dans.knaw.nl/ui/advancedsearch> |
| **Date of search** | 03-11-2023 |
| **Range of date** | None |
| **Restriction of language** | None |
| **Other limits** | None |
| **Search strategy (results)** | ('anti-infective agent*' OR 'antibiotic prophylaxis' OR 'anti-bacterial agent*' OR 'antiinfective agent*' OR 'antibiotic*' OR 'antiinfective*' OR 'anti infective*' OR 'antimicrobial' OR 'anti microbial*' OR 'antibacterial*' OR 'anti bacterial*' OR 'antibiotic* use' OR 'antimicrobial* use' OR 'antibiotic agent*' OR 'antibiotic therapy') AND ('threshold*' OR 'target*' OR 'acceptable range' OR 'cut off value*' OR 'appropriateness score' OR 'benchmarking' OR 'outcome and process assessment' OR 'goals' OR 'action plan') |
| **Number of references found** | 39 |
