# Supplementary material for: Setting targets for antibiotic use in general practice in Europe: A scoping review
Source: Eur J Gen Pract. 2024 Nov 28;30(1):2430507. doi: 10.1080/13814788.2024.2430507 (PMC11610282; doi:10.1080/13814788.2024.2430507)
Supplement: Supplemental Material [file IGEN_A_2430507_SM3297.zip › ejgp-2024-0174-File005.docx]

**Supplementary material 3. Relevant websites of infectious diseases societies, quality improvement and public health organisations**

| **Name of organisation/institution** | **Link** |
| --- | --- |
| Alliance for the Prudent Use of Antibiotics (APUA) | [www.apua.org/](http://www.apua.org/) |
| Antibiotic Centre for Primary Care | <https://www.antibiotika.no/antibiotic-centre-for-primary-care/> |
| General practice Research on Infections Network (GRIN) | <https://www.grinweb.org/> |
| European Centre for Disease Control and Prevention (ECDC) | [www.ecdc.europa.eu/](http://www.ecdc.europa.eu/) |
| European Medicines Agency (EMA) | [www.ema.europa.eu/](http://www.ema.europa.eu/) |
| European Society of Clinical Microbiology and Infectious Diseases (ESCMID) | <https://www.escmid.org/> |
| Institute for Applied Quality Improvement and Research in Health Care GmbH (AQUA) | [www.aqua-institut.de/en/home/](http://www.aqua-institut.de/en/home/) |
| International Network for the Rational Use of Drugs (INRUD) | <https://www.msh.org/journal-tags/inrud-iaa-international-network-for-the-rational-use-of-drugs-initiative-on-arv> |
| International Society of Chemotherapy Infection and Cancer (ISC) | [www.ischemo.org/](http://www.ischemo.org/) |
| International Society of Infectious Diseases | [www.isid.org/](http://www.isid.org/) |
| National Institute for Health and Care Excellence | <https://www.nice.org.uk/> |
| NHS institute for innovation and improvement | [www.institute.nhs.uk/](http://www.institute.nhs.uk/) |
| RAND Corporation | [www.rand.org/](http://www.rand.org/) |
| ReAct group | [www.reactgroup.org](http://www.reactgroup.org) |
| Swedish Strategic Programme against Antibiotic Resistance (Strama) | [www.strama.se](http://www.strama.se)  <https://strama.se/behandlingsrekommendationer/> |
| The British Society for Antimicrobial Chemotherapy (BSAC) | <http://www.bsac.org.uk/> |
| The Global Antibiotic Resistance Partnership (GARP) | [www.cddep.org/garp/home](http://www.cddep.org/garp/home) |
| Transatlantic Task Force on Antimicrobial Resistance (TATFAR) | <http://www.cdc.gov/drugresistance/tatfar/> |
| World Health Organization (WHO) | [www.who.int/](http://www.who.int/) |
| Irish College of General Practitioners (ICGP) | <https://www.icgp.ie/> |
| Royal College of General Practitioners (RCGP) | <https://www.rcgp.org.uk/> |
| WONCA Europe: World family doctors. Caring for people. | https://www.woncaeurope.org/ / https://www.woncaeurope.org/page/member-organisations |
| European General Practice Research Network (EGPRN) | <https://www.egprn.org/> |
| German Society of General Practice/Family Medicine (DEGAM) | <https://www.degam.de/> |
| Swedish Strategic Programme Against Antibiotic Resistance | <http://www.strame.se/> |
| Dutch Working Party on Antibiotic Policy (SWAB) | <http://www.swab.nl/> |
| English surveillance programme for antimicrobial utilisation and resistance (ESPAUR) report | <https://www.gov.uk/government/publications/english-surveillance-programme-antimicrobial-utilisation-and-resistance-espaur-report> |
| Spanish National Plan against Antibiotic Resistance (PRAN) | <https://www.resistenciaantibioticos.es/es/quienes-somos> |
| Collection: Antimicrobial resistance (AMR) (UK government) | <https://www.gov.uk/government/collections/antimicrobial-resistance-amr-information-and-resources> |
| AMR local indicators - produced by the UKHSA | <https://fingertips.phe.org.uk/profile/amr-local-indicators> |
| Train-the-Trainer Event: Defining and Implementing Responsible Antibiotic Use | <http://drive-ab.eu/events/train-the-trainer-event-defining-and-implementing-responsible-antibiotic-use/> |
