# Supplementary material for: Setting targets for antibiotic use in general practice in Europe: A scoping review
Source: Eur J Gen Pract. 2024 Nov 28;30(1):2430507. doi: 10.1080/13814788.2024.2430507 (PMC11610282; doi:10.1080/13814788.2024.2430507)
Supplement: Supplemental Material [file IGEN_A_2430507_SM3297.zip › ejgp-2024-0174-File006.docx]

# Supplementary material 4. List of reports excluded and reasons for their exclusion

| **n** | **ID record** | **Title** | **Reason for exclusion** |
| --- | --- | --- | --- |
| ^1^ | Rautakorpi 2001 | Antibiotic use by indication: a basis for active antibiotic policy in the community | Not targets for antibiotic use |
| ^2^ | Ranji 2006 | Closing the Quality Gap: A Critical Analysis of Quality Improvement Strategies | Not target for antibiotic use, it focused on clinician prescribing behavior |
| ^3^ | Carelli 2023 | The best laid plans?: international governance perspectives in AMR national action plans in Europe | Not target for antibiotic use and not general practice |
| ^4^ | Jones 2020 | Self-Reported Antimicrobial Stewardship Practices in Primary Care Using the TARGET Antibiotics Self-Assessment Tool | Not targets for antibiotic use |
| ^5^ | Exner 2020 | Prescribing of antibiotics for respiratory tract infections in German outpatient pediatric care: Results of a survey of pediatricians and general practitioners | Not targets for antibiotic use |
| ^6^ | Tsopra 2019 | Helping GPs to extrapolate guideline recommendations to patients for whom there are no explicit recommendations, through the visualization of drug properties. The example of AntibioHelp R in bacterial diseases | Not target for antibiotic use, it focused on explore clinical recommendations for antibiotic prescribing |
| ^7^ | Schierenberg 2019 | Antibiotic treatment of gastroenteritis in primary care | Not target for antibiotic use, it focused on explore targeted treatment and clinical recommendations for antibiotic prescribing |
| ^8^ | LeMarechal 2018 | Quality indicators assessing antibiotic use in the outpatient setting: a systematic review followed by an international multidisciplinary consensus procedure | Not target for antibiotic use, Systematic review was focused on developing a list of generic quality indicators that can be assessed and adapted to national specificities and guidelines |
| ^9^ | Davies 2018 | Reducing inappropriate prescribing of antibiotics in English primary care: evidence and outlook | Opinion paper |
| ^10^ | Zweigner 2018 | Rate of antibiotic prescriptions in German outpatient care - are the guidelines followed or are they still exceeded? | Not targets for antibiotic use |
| ^11^ | Pulcini 2013 | Drug-specific quality indicators assessing outpatient antibiotic use among French general practitioners | Not target for antibiotic use, it focused on quality indicator |
| ^12^ | Filippini 2013 | Assessing the impact of national antibiotic campaigns in Europe | Not targets for antibiotic use |
| ^13^ | Wood 2011 | Antibiotic prescribing for adults with acute cough/lower respiratory tract infection: congruence with guidelines | Not target, it focused on exploring congruence with guideline recommendations |
| ^14^ | Boesten 2011 | Defining antimicrobial prescribing quality indicators: what is a new prescription? | Not target for antibiotic use, it focused on quality indicator |
| ^15^ | Gunnlaugsdottir 2021 | Encouraging rational antibiotic prescribing behaviour in primary care–prescribing practice among children aged 0–4 years 2016–2018: an observational study | Not targets for antibiotic use |
| ^16^ | Castro-Sánchez 2018 | Articulating citizen participation in national anti-microbial resistance plans: A comparison of European countries | Not target for antibiotic use and not general practice |
| ^17^ | Bourdellon 2016 | Impact and acceptance of the antibiogram targeted on the prescription of antibiotics in general medicine throughout the French region | Conference abstracts |
| ^18^ | Versporten 2014 | Antibiotic use in eastern Europe: A cross-national datantibioticase study in coordination with the WHO Regional Office for Europe | Not targets for antibiotic use |
| ^19^ | Juhasz 2013 | Treatment of acute cystitis in Hungary: Comparison with national guidelines and with disease-specific quality indicators | Not targets for antibiotic use and all prescriptions claimed in the community pharmacies of Hungary |
| ^20^ | Maciulaitis 2006 | Assessment of antibiotic use and comparison with recommendations for their rational use | Not target for antibiotic use, it focused on explore clinical recommendations for antibiotic prescribing |
| ^21^ | Thompson 2022 | An audit of acute respiratory antibiotic prescribing in COPD patients during the COVID-19 pandemic | Conference abstracts |
| ^22^ | André 2018 | Quality indicators aiming to help primary care in Sweden to balance between what to do and what not to do quality indicators aiming to help primary care in sweden to balance between what to do and what not to do | Conference abstracts |
| ^23^ | Kaguelidou 2016 | New quality indicators for paediatric antibiotic prescribing in primary care: a population based cohort study in the United Kingdom, Italy and the Netherlands from 1995-2010 | Conference abstracts |
| ^24^ | Ashiru-Oredope 2015 | Evaluating the implementation of national antimicrobial stewardship toolkits for primary and secondary care in England | Conference abstracts |
| ^25^ | Hurding 2015 | Patient Safety through National Therapeutic Indicators | Conference abstracts |
| ^26^ | Ribeirinho 2013 | Antimicrobial utilization in hospital and primary care in Portugal mainland | Conference abstracts |
| ^27^ | Adriaenssens 2013 | Feasibility and outcome of applying disease-specific antibiotic prescribing quality indicators | Conference abstracts |
| ^28^ | Pulcini 2012 | Drug-specific indicators assessing outpatient antibiotic use: A cross-sectional study of French general practitioners | Conference abstracts |
| ^29^ | Sneddon 2011 | Development of national prescribing indicators for antimicrobials to support reduction in Clostridium difficile infection | Conference abstracts |
| ^30^ | Zahra 2010 | Supporting GPs and PBC groups to reduce antibiotic usage | Conference abstracts |
| ^31^ | Wang 2008 | Beware prescribing targets and guides | Conference abstracts |
| ^32^ | Reingold 2023 | Pediatric antibiotic stewardship programs in Europe: a pilot survey among delegates of The European Academy of Pediatrics | Not target for antibiotic use and not general practice |
| ^33^ | Fernández-Urrusuno 2020 | Successful improvement of antibiotic prescribing at Primary Care in Andalusia following the implementation of an antimicrobial guide through multifaceted interventions: An interrupted time-series analysis | Not general practice, only one center (pharmacy) incorporation of targets for antibiotic prescribing indicators linked to financial incentives |
| ^34^ | Adriaenssens 2021 | Quality appraisal of antibiotic consumption in the community, European Union/European Economic Area, 2009 and 2017 | Not target for antibiotic use and not general practice |
| ^35^ | Sharland 2022 | The WHO essential medicines list AWaRe book: from a list to a quality improvement system | Commentary |
| ^36^ | Courtenay 2019 | Tackling antimicrobial resistance 2019–2024 – The UK's five-year national action plan | Editorial |
| ^37^ | Avent 2020 | Antimicrobial stewardship in the primary care setting: from dream to reality? | Not targets for antibiotic use |
| ^38^ | Dekker 2015 | Inappropriate antibiotic prescription for respiratory tract indications: Most prominent in adult patients | Not targets for antibiotic use |
| ^39^ | van der Velden 2016 | Usefulness of quality indicators for antibiotic use: case study for the Netherlands | Not target for antibiotic use, it focused on quality indicator |
| ^40^ | van der Velden 2020 | Structural antibiotic surveillance and stewardship via indication-linked quality indicators: Pilot in dutch primary care | Not target for antibiotic use, it focused on quality indicator |
| ^41^ | Hawes 2020 | Antimicrobial stewardship in general practice: A scoping review of the component parts | Not target for antibiotic use, review was focused on national action plan analysis |
| ^42^ | Delory 2020 | A computerized decision support system (CDSS) for antibiotic prescription in primary care-Antibioclic: implementation, adoption and sustainantibioticle use in the era of extended antimicrobial resistance | Not target, it focused to monitors official updates of national guidelines for each pathology/infectious disease |
| ^43^ | Saust 2016 | Quality assessment of diagnosis and antibiotic treatment of infectious diseases in primary care: a systematic review of quality indicators | Not target for antibiotic use, it focused on quality indicator |
| ^44^ | Mitsakakis 2018 | Challenges in identifying antibiotic resistance targets for point-of-care diagnostics in general practice | Opinion paper |
| ^45^ | Chang 2021 | European Respiratory Society guidelines for the management of children and adolescents with bronchiectasis | Not target for antibiotic use, it focused on develop a guideline for managing children and adolescents with bronchiectasis unrelated to cystic fibrosis |
| ^46^ | Allison 2020 | What resources do NHS commissioning organisations use to support antimicrobial stewardship in primary care in England? | Not targets for antibiotic use |
| ^47^ | Ashiru-Oredope 2018 | Antimicrobial stewardship programmes in community healthcare organisations in England: A cross-sectional survey to assess implementation of programmes and national toolkits | Not targets for antibiotic use |
| ^48^ | Mauffrey 2016 | Perception of acceptantibioticle antibiotic stewardship strategies in outpatient settings | Not targets for antibiotic use |
| ^49^ | Alves 2021 | Antibiotic prescribing in UK out-of-hours primary care services: a realist-informed scoping review of training and guidelines for healthcare professionals | Not target for antibiotic use, it focused on explore clinical recommendations for antibiotic prescribing |
| ^50^ | Aghlmandi 2023 | Effect of Antibiotic Prescription Audit and Feedback on Antibiotic Prescribing in Primary Care: A Randomized Clinical Trial | Not targets for antibiotic use |
| ^51^ | Watier 2017 | Comparing antibiotic consumption between two European countries: are packages an adequate surrogate for prescriptions? | Not target for antibiotic use and not general practice |
| ^52^ | Atkins 2020 | Content and mechanism of action of national antimicrobial stewardship interventions on management of respiratory tract infections in primary and community care | Not targets for antibiotic use |
| ^53^ | Poss-Doering 2021 | Antibiotic prescribing for acute, non-complicated infections in primary care in Germany: baseline assessment in the cluster randomized trial ARena | Not targets for antibiotic use |
| ^54^ | Palin 2019 | Antibiotic prescribing for common infections in UK general practice: variantibioticility and drivers | Not targets for antibioitc use, it focused on exploring congruence with guideline recommendations |
| ^55^ | Sijbom 2023 | Determinants of inappropriate antibiotic prescription in primary care in developed countries with general practitioners as gatekeepers: A systematic review and construction of a framework | Not targets for antibiotic use |
| ^56^ | Hummers-Pradier 2005 | Management of urinary tract infections in female general practice patients | Not targets for antibiotic use |
| ^57^ | Allison 2020 | Local implementation of national guidance on management of common infections in primary care in England | Not targets for antibiotic use |
| ^58^ | Muijrers 2004 | Prescribing indicators. Development and validation of guideline-based prescribing indicators as an instrument to measure the variation in the prescribing behaviour of general practitioners | Not targets for antibiotic use |
| ^59^ | Bennett Institute for Applied Data Science 2024 | Openprescribing: Explore England's prescribing data | Not targets for antibiotic use in the dashboard |
| ^60^ | Degnan 2022 | Development and validation of antibiotic stewardship metrics for outpatient respiratory tract diagnoses and association of provider characteristics with inappropriate prescribing | Not targets for antibiotic use |
| ^61^ | Kern 2021 | Organization of antibiotic stewardship in Europe: the way to go | Unclear setting (general practices) |
| ^62^ | Kopsidas 2020 | A Survey on National Pediatric Antibiotic Stewardship Programs, Networks and Guidelines in 23 European Countries | Not targets for antibiotic use |
| ^63^ | Buehrle 2020 | Sustained Reductions in Overall and Unnecessary Antibiotic Prescribing at Primary Care Clinics in a Veterans Affairs Healthcare System Following a Multifaceted Stewardship Intervention | Set in America and did not mention targets |
| ^64^ | Schuts 2016 | Adoption of a national antimicrobial guide (SWAB-ID) in the Netherlands | Not targets for antibiotic use |
| ^65^ | Fernandez-Urrusuno 2015 | Selection of indicators for continuous monitoring of the impact of programs optimizing antimicrobial use in Primary Care | Not targets for antibiotic use |
| ^66^ | Rojo-Martín 2018 | The PIRASOA programme: design, structure, organisation and indicators of a comprehensive regional Institutional Programme for the Prevention and Control of Healthcare-associated Infections and Antimicrobial Stewardship for hospitals and primary care settings in Andalusia, Spain V.2 | Not targets for antibiotic use, it focused on quality indicator |
| ^67^ | Fernandez-Urrusuno 2013 | Compliance with quality prescribing indicators in terms of their relationship to financial incentives | Not targets for antibiotic use |
| ^68^ | Sanchez 2016 | Core Elements of Outpatient Antibiotic Stewardship | Not targets for antibiotic use |
| ^69^ | World Health Organization 2015 | Global action plan on antimicrobial resistance. | Not targets for antibiotic use, it is global national action plan |
| ^70^ | Busse 2019 | Improving healthcare quality in Europe: Characteristics, effectiveness and implementation of different strategies | Not targets for antibiotic use |
| ^71^ | Health Prevention 2017 | PNCAR—National action plan on antimicrobial resistance. | Setting is not general practice |
| ^72^ | Zoorob 2012 | Antibiotic Use in Acute Upper Respiratory Tract Infections | Not targets for antibiotic use |
| ^73^ | van den Broek d'Obrenan 2014 | Antibiotic use in Dutch primary care: relation between diagnosis, consultation and treatment | Not targets for antibiotic use |
| ^74^ | O’Connor 2020 | Improving the quality of antibiotic prescribing through an educational intervention delivered through the out-of-hours general practice service in Ireland | Not targets for antibiotic use |
| ^75^ | European Centre for Disease Prevention and Control 2022 | Antimicrobial consumption in the EU/EEA (ESAC-Net) - Annual Epidemiological Report for 2021 | Setting is not general practice |
| ^76^ | European Centre for Disease Prevention and Control 2014 | Surveillance of antimicrobial consumption in Europe 2012 | Not targets for antibiotic use |
| ^77^ | DePestel 2014 | Assessing appropriateness of antimicrobial therapy: in the eye of the interpreter | Not targets for antibiotic use and based in USA |
| ^78^ | van den Bosch 2015 | Quality Indicators to Measure Appropriate Antibiotic Use in Hospitalized Adults | No targets only quality indicator mentioned and hospital setting |
| ^79^ | Monnier 2018 | Quality indicators for responsible antibiotic use in the inpatient setting: a systematic review followed by an international multidisciplinary consensus procedure | Not targets for antibiotic use |
| ^80^ | Goossens 2006 | National campaigns to improve antibiotic use | Not targets for antibiotic use |
| ^81^ | ECDC EFSA Panel on Biological Hazards (BIOHAZ) 2017 | ECDC, EFSA and EMA Joint Scientific Opinion on a list ofoutcome indicators as regards surveillance of antimicrobialresistance and antimicrobial consumption in humans andfood-producing animals | Setting is not general practice |
| ^82^ | Charra 2019 | Impact of amoxicillin and oral amoxicillin-clavulanic acid defined daily doses on consumption indicators | No targets mentioned |
| ^83^ | Glinz 2017 | Quality of antibiotic prescribing of Swiss primary care physicians with high prescription rates: a nationwide survey | Not targets for antibiotic use |
| ^84^ | Ivanovska 2016 | Antibiotic prescribing for children in primary care and adherence to treatment guidelines | Not targets for antibiotic use |
| ^85^ | Balinskaite 2018 | The Impact of a National Antimicrobial Stewardship Program on Antibiotic Prescribing in Primary Care: An Interrupted Time Series Analysis | Not targets for antibiotic use |
| ^86^ | Coenen 2014 | Appropriate international measures for outpatient antibiotic prescribing and consumption: recommendations from a national data comparison of different measures | Setting is not general practice |
| ^87^ | Sabuncu 2009 | Significant Reduction of Antibiotic Use in the Community after a Nationwide Campaign in France, 2002–2007 | Not targets for antibiotic use |
| ^88^ | Sanchez 2016a | The Core Elements of Outpatient Antibiotic Stewardship | Not targets for antibiotic use |
| ^89^ | Ashiru-Oredope 2013 | Antimicrobial stewardship: English Surveillance Programme for Antimicrobial Utilization and Resistance (ESPAUR) | Not targets for antibiotic use |
| ^90^ | McNulty 2001 | Optimising antibiotic prescribing in primary care | Not targets for antibiotic use |
| ^91^ | Ashiru-Oredope 2012 | Improving the quality of antibiotic prescribing in the NHS by developing a new Antimicrobial Stewardship Programme: Start Smart--Then Focus | Not targets for antibiotic use |
| ^92^ | Llor 2014 | Antimicrobial resistance: risk associated with antibiotic overuse and initiatives to reduce the problem | Not targets for antibiotic use |
| ^93^ | UK Health Security Agency 2022 | English surveillance programme for antimicrobial utilisation and resistance (ESPAUR) | Not targets for antibiotic use |
| ^94^ | Department of Health and Social Care 2019 | Contained and controlled The UK’s 20-year vision for antimicrobial resistance | Setting is not general practice |
| ^95^ | Williams 2018 | Antibiotic prescribing quality for children in primary care: an observational study | Not targets for antibiotic use |
| ^96^ | Hedin 2014 | Management of patients with sore throats in relation to guidelines: an interview study in Sweden | Not targets for antibiotic use |
| ^97^ | Jim 2016 | Tackling drug-resistant infections globally: final report and recommendations | Setting is not general practice |
| ^98^ | Smith 2018 | Defining the appropriateness and inappropriateness of antibiotic prescribing in primary care | Not targets for antibiotic use |
| ^99^ | Bateman 1996 | Setting standards of prescribing performance in primary care: use of a consensus group of general practitioners and application of standards to practices in the north of England | Not targets for antibiotic use |
| ^100^ | Johnson 2015 | Surveillance of antibiotic resistance | Setting is not general practice |
| ^101^ | Kronman 2020 | Reducing Antibiotic Prescribing in Primary Care for Respiratory Illness | Not targets for antibiotic use |
| ^102^ | Santos 2019 | Interventions to reduce the prescription of inappropriate medicines in older patients | Not targets for antibiotic use |
| ^103^ | Nathwani 2012 | Antimicrobial stewardship in Scotland: impact of a national programme | Target not specific to general practice |
| ^104^ | Hawker 2014 | Trends in antibiotic prescribing in primary care for clinical syndromes subject to national recommendations to reduce antibiotic resistance, UK 1995-2011: analysis of a large database of primary care consultations | Not targets for antibiotic use |
| ^105^ | Rose 2021 | A qualitative literature review exploring the drivers influencing antibiotic over-prescribing by GPs in primary care and recommendations to reduce unnecessary prescribing | Not targets for antibiotic use |
| ^106^ | Malmros 2019 | Comparison of antibiotic treatment guidelines for urinary tract infections in 15 European countries: Results of an online survey | Not targets for antibiotic use |
| ^107^ | Fernandez 2014 | Improving the appropriateness of antimicrobial use in primary care after implementation of a local antimicrobial guide in both levels of care | Not targets for antibiotic use |
| ^108^ | Ashworth 2002 | Prescribing indicators and their use by primary care groups to influence prescribing. | It was not clear the specific target for antibiotics |
| ^109^ | Ashworth 2004 | How are primary care organizations using financial incentives to influence prescribing? | It was not clear the specific target for antibiotics |

**References**

1. Rautakorpi UM, Klaukka T, Honkanen P, et al. Antibiotic use by indication: a basis for active antibiotic policy in the community. *Scandinavian journal of infectious diseases* 2001;33(12):920-26.

2. Ranji SR, Steinman MA, Shojania KG, et al. Closing the Quality Gap: A Critical Analysis of Quality Improvement Strategies (Vol. 4: Antibiotic Prescribing Behavior). 2006

3. Carelli DE, Mitsouli ET, Ogne JB, Pierre J. The best laid plans?: international governance perspectives in AMR national action plans in Europe. *European journal of public health* 2023;33(4):682-86. doi: <https://dx.doi.org/10.1093/eurpub/ckad080> PT - Journal Article, Research Support, Non-U.S. Gov't

4. Jones LF, Verlander NQ, Lecky DM, et al. Self-Reported Antimicrobial Stewardship Practices in Primary Care Using the TARGET Antibiotics Self-Assessment Tool. *Antibiotics (Basel, Switzerland)* 2020;9(5) doi: <https://dx.doi.org/10.3390/antibiotics9050253> PT - Journal Article

5. Exner V, Hoser C, Trapp S, Simon A. Prescribing of antibiotics for respiratory tract infections in German outpatient pediatric care : Results of a survey of pediatricians and general practitioners. *Antibiotikaverordnungen bei Atemwegsinfektionen im Kindesalter : Eine bundesweite Umfrage bei Facharzten fur Kinder- und Jugendmedizin sowie Allgemeinmedizin* 2020;63(10):1231-40. doi: <https://dx.doi.org/10.1007/s00103-020-03214-8> PT - Journal Article

6. Tsopra R, Sedki K, Courtine M, et al. Helping GPs to extrapolate guideline recommendations to patients for whom there are no explicit recommendations, through the visualization of drug properties. The example of AntibioHelp R in bacterial diseases. *Journal of the American Medical Informatics Association : JAMIA* 2019;26(10):1010-19. doi: <https://dx.doi.org/10.1093/jamia/ocz057> PT - Journal Article, Research Support, Non-U.S. Gov't

7. Schierenberg A, Bruijning-Verhagen PCJ, van Delft S, et al. Antibiotic treatment of gastroenteritis in primary care. *The Journal of antimicrobial chemotherapy* 2019;74(1):207-13. doi: <https://dx.doi.org/10.1093/jac/dky385> PT - Journal Article

8. Le Marechal M, Tebano G, Monnier AA, et al. Quality indicators assessing antibiotic use in the outpatient setting: a systematic review followed by an international multidisciplinary consensus procedure. *The Journal of antimicrobial chemotherapy* 2018;73(suppl_6):vi40-vi49. doi: <https://dx.doi.org/10.1093/jac/dky117> PT - Journal Article, Research Support, Non-U.S. Gov't, Systematic Review

9. Davies SC. Reducing inappropriate prescribing of antibiotics in English primary care: evidence and outlook. *The Journal of antimicrobial chemotherapy* 2018;73(4):833-34. doi: <https://dx.doi.org/10.1093/jac/dkx535> PT - Journal Article

10. Zweigner J, Meyer E, Gastmeier P, Schwab F. Rate of antibiotic prescriptions in German outpatient care - are the guidelines followed or are they still exceeded? *GMS hygiene and infection control* 2018;13:Doc04-Doc04. doi: <https://dx.doi.org/10.3205/dgkh000310> PT - Journal Article

11. Pulcini C, Lions C, Ventelou B, Verger P. Drug-specific quality indicators assessing outpatient antibiotic use among French general practitioners. *European journal of public health* 2013;23(2):262-64. doi: <https://dx.doi.org/10.1093/eurpub/cks100> PT - Journal Article, Research Support, Non-U.S. Gov't

12. Filippini M, Ortiz LGG, Masiero G. Assessing the impact of national antibiotic campaigns in Europe. *The European journal of health economics : HEPAC : health economics in prevention and care* 2013;14(4):587-99. doi: <https://dx.doi.org/10.1007/s10198-012-0404-9> PT - Journal Article

13. Wood J, Butler CC, Hood K, et al. Antibiotic prescribing for adults with acute cough/lower respiratory tract infection: congruence with guidelines. *The European respiratory journal* 2011;38(1):112-18. doi: <https://dx.doi.org/10.1183/09031936.00145810> PT - Journal Article, Multicenter Study, Research Support, Non-U.S. Gov't

14. Boesten J, Harings L, Winkens B, et al. Defining antimicrobial prescribing quality indicators: what is a new prescription? *European journal of clinical pharmacology* 2011;67(1):91-96. doi: <https://dx.doi.org/10.1007/s00228-010-0909-z> PT - Journal Article

15. Gunnlaugsdottir MR, Linnet K, Jonsson JS, Blondal AB. Encouraging rational antibiotic prescribing behaviour in primary care–prescribing practice among children aged 0–4 years 2016–2018: an observational study. *Scandinavian Journal of Primary Health Care* 2021;39(3):373-81. doi: 10.1080/02813432.2021.1958506

16. Castro-Sánchez E, Iwami M, Ahmad R, et al. Articulating citizen participation in national anti-microbial resistance plans: A comparison of European countries. *European Journal of Public Health* 2018;28(5):928-34. doi: 10.1093/eurpub/cky128

17. Bourdellon L, Pulcini C, Fougnot S, et al. Impact and acceptance of the antibiogram targeted on the prescription of antibiotics in general medicine throughout the French region. *Medecine et Maladies Infectieuses* 2016;46(4):1-1. doi: 10.1016/S0399-077X(16)30257-8

18. Versporten A, Bolokhovets G, Ghazaryan L, et al. Antibiotic use in eastern Europe: A cross-national database study in coordination with the WHO Regional Office for Europe. *The Lancet Infectious Diseases* 2014;14(5):381-87. doi: 10.1016/S1473-3099(14)70071-4

19. Juhasz Z, Benko R, Matuz M, et al. Treatment of acute cystitis in Hungary: Comparison with national guidelines and with disease-specific quality indicators. *Scandinavian Journal of Infectious Diseases* 2013;45(8):612-15. doi: 10.3109/00365548.2013.777157

20. Maciulaitis R, Janusonis T, Petrikaite V, Aukstakalniene A. Assessment of antibiotic use and comparison with recommendations for their rational use. *Medicina (Kaunas, Lithuania)* 2006;42(12):999-1005.

21. Thompson J, Widdows G, Parbat M. An audit of acute respiratory antibiotic prescribing in COPD patients during the COVID-19 pandemic. *International Journal of Pharmacy Practice* 2022;30(SUPPL 1):i24-i24. doi: 10.1093/ijpp/riac019.033 LK - <http://jq6am9xs3s.search.serialssolutions.com/?sid=EMBASE&issn=20427174&id=doi:10.1093%2Fijpp%2Friac019.033&atitle=AN+AUDIT+OF+ACUTE+RESPIRATORY+ANTIBIOTIC+PRESCRIBING+IN+COPD+PATIENTS+DURING+THE+COVID-19+PANDEMIC&stitle=Int>

22. André M, Elmroth U, Arvidsson E, Månsson J. Quality indicators aiming to help primary care in sweden to balance between what to do and what not to doquality indicators aiming to help primary care in sweden to balance between what to do and what not to do. *BMJ Evidence-Based Medicine* 2018;23:A40-A40. doi: 10.1136/bmjebm-2018-111070.86 LK - <http://jq6am9xs3s.search.serialssolutions.com/?sid=EMBASE&issn=25154478&id=doi:10.1136%2Fbmjebm-2018-111070.86&atitle=Quality+indicators+aiming+to+help+primary+care+in+sweden+to+balance+between+what+to+do+and+what+not+t>

23. Kaguelidou F, De Bie S, Verhamme K, et al. New quality indicators for paediatric antibiotic prescribing in primary care: a population based cohort study in the United Kingdom, Italy and the Netherlands from 1995-2010. *Archives of Disease in Childhood* 2016;101(1):3-4. doi: 10.1136/archdischild-2015-310148.9 LK - <http://jq6am9xs3s.search.serialssolutions.com/?sid=EMBASE&issn=14682044&id=doi:10.1136%2Farchdischild-2015-310148.9&atitle=NEW+QUALITY+INDICATORS+for+PAEDIATRIC+ANTIBIOTIC+PRESCRIBING+in+PRIMARY+CARE%3A+A+POPULATIO>

24. Ashiru-Oredope DA, Budd E, Beech E, et al. Evaluating the implementation of national antimicrobial stewardship toolkits for primary and secondary care in England. *International Journal of Pharmacy Practice* 2015;23:15-15. doi: 10.1111/ijpp.12211 LK - <http://jq6am9xs3s.search.serialssolutions.com/?sid=EMBASE&issn=09617671&id=doi:10.1111%2Fijpp.12211&atitle=Evaluating+the+implementation+of+national+antimicrobial+stewardship+toolkits+for+primary+and+secondary+care+in+England&stit>

25. Hurding S, MacBride-Stewart S, Scott B, et al. Patient Safety through National Therapeutic Indicators. *International Journal of Clinical Pharmacy* 2015;37(1):280-80. doi: 10.1007/s11096-014-0039-2 LK - <http://jq6am9xs3s.search.serialssolutions.com/?sid=EMBASE&issn=22107703&id=doi:10.1007%2Fs11096-014-0039-2&atitle=Patient+Safety+through+National+Therapeutic+Indicators&stitle=Int.+J.+Clin.+Pharm.&title=International+Journa>

26. Ribeirinho M. Antimicrobial utilization in hospital and primary care in Portugal mainland. *Pharmacoepidemiology and Drug Safety* 2013;22:491-91. doi: 10.1002/pds.3512 LK - <http://jq6am9xs3s.search.serialssolutions.com/?sid=EMBASE&issn=10538569&id=doi:10.1002%2Fpds.3512&atitle=Antimicrobial+utilization+in+hospital+and+primary+care+in+Portugal+mainland&stitle=Pharmacoepidemiol.+Drug+Saf.&title=Pharmacoe>

27. Adriaenssens N, Bartholomeeusen S, Ryckebosch P, Coenen S. Feasibility and outcome of applying disease-specific antibiotic prescribing quality indicators. *European Journal of General Practice* 2013;19(1):35-35. doi: 10.3109/13814788.2012.759936 LK - <http://jq6am9xs3s.search.serialssolutions.com/?sid=EMBASE&issn=13814788&id=doi:10.3109%2F13814788.2012.759936&atitle=Feasibility+and+outcome+of+applying+disease-specific+antibiotic+prescribing+quality+indicators&stitle=E>

28. Pulcini C, Lions C, Ventelou B, Verger P. Drug-specific indicators assessing outpatient antibiotic use: A cross-sectional study of French general practitioners. *Clinical Microbiology and Infection* 2012;18:765-65. doi: 10.1111/j.1469-0691.2012.03802.x LK - <http://jq6am9xs3s.search.serialssolutions.com/?sid=EMBASE&issn=1198743X&id=doi:10.1111%2Fj.1469-0691.2012.03802.x&atitle=Drug-specific+indicators+assessing+outpatient+antibiotic+use%3A+A+cross-sectional+study+of+Fren>

29. Sneddon J, Nathwani D, Patton A, et al. Development of national prescribing indicators for antimicrobials to support reduction in Clostridium difficile infection. *Clinical Microbiology and Infection* 2011;17:S7-S7. doi: 10.1111/j.1469-0691.2011.03557.x LK - <http://jq6am9xs3s.search.serialssolutions.com/?sid=EMBASE&issn=1198743X&id=doi:10.1111%2Fj.1469-0691.2011.03557.x&atitle=Development+of+national+prescribing+indicators+for+antimicrobials+to+support+reduction+in+Clost>

30. Zahra I, Alison S, Carol C, et al. Supporting GPs and PBC groups to reduce antibiotic usage. *Pharmacoepidemiology and Drug Safety* 2010;19(6):651-51. doi: 10.1002/pds LK - <http://jq6am9xs3s.search.serialssolutions.com/?sid=EMBASE&issn=10538569&id=doi:10.1002%2Fpds&atitle=Supporting+GPs+and+PBC+groups+to+reduce+antibiotic+usage&stitle=Pharmacoepidemiol.+Drug+Saf.&title=Pharmacoepidemiology+and+Drug+Safety&v>

31. Wang LN. Beware prescribing targets and guides. *Pharmaceutical Journal* 2008;280(7494):343-43.

32. Reingold SM, Grossman Z, Hadjipanayis A, et al. Pediatric antibiotic stewardship programs in Europe: a pilot survey among delegates of The European Academy of Pediatrics. *Frontiers in Pediatrics* 2023;11 doi: 10.3389/fped.2023.1157542 LK - <http://jq6am9xs3s.search.serialssolutions.com/?sid=EMBASE&issn=22962360&id=doi:10.3389%2Ffped.2023.1157542&atitle=Pediatric+antibiotic+stewardship+programs+in+Europe%3A+a+pilot+survey+among+delegates+of+The+European+Academy>

33. Fernández-Urrusuno R, Meseguer Barros CM, Benavente Cantalejo RS, et al. Successful improvement of antibiotic prescribing at Primary Care in Andalusia following the implementation of an antimicrobial guide through multifaceted interventions: An interrupted time-series analysis. *PLoS ONE* 2020;15(5):e0233062-e62. doi: <https://dx.doi.org/10.1371/journal.pone.0233062> PT - Journal Article, Research Support, Non-U.S. Gov't

34. Adriaenssens N, Bruyndonckx R, Versporten A, et al. Quality appraisal of antibiotic consumption in the community, European Union/European Economic Area, 2009 and 2017. *Journal of Antimicrobial Chemotherapy* 2021;76:II60-II67. doi: 10.1093/jac/dkab178 LK - <http://jq6am9xs3s.search.serialssolutions.com/?sid=EMBASE&issn=14602091&id=doi:10.1093%2Fjac%2Fdkab178&atitle=Quality+appraisal+of+antibiotic+consumption+in+the+community%2C+European+Union%2FEuropean+Economic+Area%2C+2009+and+201>

35. Sharland M, Zanichelli V, Ombajo LA, et al. The WHO essential medicines list AWaRe book: from a list to a quality improvement system. *Clinical Microbiology and Infection* 2022;28(12):1533-35. doi: 10.1016/j.cmi.2022.08.009

36. Courtenay M, Castro-Sanchez E, Fitzpatrick M, et al. Tackling antimicrobial resistance 2019–2024 – The UK's five-year national action plan. *Journal of Hospital Infection* 2019;101(4):426-27. doi: 10.1016/j.jhin.2019.02.019 LK - <http://jq6am9xs3s.search.serialssolutions.com/?sid=EMBASE&issn=15322939&id=doi:10.1016%2Fj.jhin.2019.02.019&atitle=Tackling+antimicrobial+resistance+2019%E2%80%932024+%E2%80%93+The+UK%27s+five-year+national+action+plan&sti>

37. Avent ML, Cosgrove SE, Price-Haywood EG, van Driel ML. Antimicrobial stewardship in the primary care setting: from dream to reality? *BMC family practice* 2020;21(1):134-34. doi: 10.1186/s12875-020-01191-0 LK - <http://jq6am9xs3s.search.serialssolutions.com/?sid=EMBASE&issn=14712296&id=doi:10.1186%2Fs12875-020-01191-0&atitle=Antimicrobial+stewardship+in+the+primary+care+setting%3A+from+dream+to+reality%3F&stitle=BMC+Fam+Pract&titl>

38. Dekker ARJ, Verheij TJM, van der Velden AW. Inappropriate antibiotic prescription for respiratory tract indications: Most prominent in adult patients. *Family practice* 2015;32(4):401-07. doi: <https://dx.doi.org/10.1093/fampra/cmv019> PT - Journal Article, Multicenter Study, Observational Study, Research Support, Non-U.S. Gov't

39. van der Velden AW, Roukens M, van de Garde E, et al. Usefulness of quality indicators for antibiotic use: case study for the Netherlands. *International Journal for Quality in Health Care* 2016;28(6):838-42. doi: <https://dx.doi.org/10.1093/intqhc/mzw117> PT - Journal Article

40. van der Velden AW, van Triest MI, Schoffelen AF, Verheij TJM. Structural antibiotic surveillance and stewardship via indication-linked quality indicators: Pilot in dutch primary care. *Antibiotics* 2020;9(10):1-11. doi: 10.3390/antibiotics9100670

41. Hawes L, Buising K, Mazza D. Antimicrobial stewardship in general practice: A scoping review of the component parts. *Antibiotics* 2020;9(8):1-13. doi: 10.3390/antibiotics9080498

42. Delory T, Jeanmougin P, Lariven S, et al. A computerized decision support system (CDSS) for antibiotic prescription in primary care-Antibioclic: implementation, adoption and sustainable use in the era of extended antimicrobial resistance. *The Journal of antimicrobial chemotherapy* 2020;75(8):2353-62. doi: 10.1093/jac/dkaa167 LK - <http://jq6am9xs3s.search.serialssolutions.com/?sid=EMBASE&issn=14602091&id=doi:10.1093%2Fjac%2Fdkaa167&atitle=A+computerized+decision+support+system+%28CDSS%29+for+antibiotic+prescription+in+primary+care-Antibioclic%3A+implementa>

43. Saust LT, Monrad RN, Hansen MP, et al. Quality assessment of diagnosis and antibiotic treatment of infectious diseases in primary care: a systematic review of quality indicators. *Scandinavian Journal of Primary Health Care* 2016;34(3):258-66. doi: 10.1080/02813432.2016.1207143

44. Mitsakakis K, Kaman WE, Elshout G, et al. Challenges in identifying antibiotic resistance targets for point-of-care diagnostics in general practice. *Future Microbiology* 2018;13(10):1157-64. doi: 10.2217/fmb-2018-0084

45. Chang AB, Fortescue R, Grimwood K, et al. European Respiratory Society guidelines for the management of children and adolescents with bronchiectasis. *The European respiratory journal* 2021;58(2) doi: 10.1183/13993003.02990-2020

46. Allison R, Lecky DM, Beech E, et al. What resources do NHS commissioning organisations use to support antimicrobial stewardship in primary care in England? *Antibiotics* 2020;9(4) doi: <https://dx.doi.org/10.3390/antibiotics9040158> PT - Journal Article

47. Ashiru-Oredope D, Doble A, Akpan MR, et al. Antimicrobial stewardship programmes in community healthcare organisations in England: A cross-sectional survey to assess implementation of programmes and national toolkits. *Antibiotics* 2018;7(4) doi: <https://dx.doi.org/10.3390/antibiotics7040097> PT - Journal Article

48. Mauffrey V, Kivits J, Pulcini C, Boivin JM. Perception of acceptable antibiotic stewardship strategies in outpatient settings. *Medecine et Maladies Infectieuses* 2016;46(6):285-93. doi: <https://dx.doi.org/10.1016/j.medmal.2016.06.006> PT - Journal Article

49. Alves PG, Hayward G, Leydon G, et al. Antibiotic prescribing in UK out-of-hours primary care services: a realist-informed scoping review of training and guidelines for healthcare professionals. *BJGP Open* 2021;5(3):1-9. doi: 10.3399/BJGPO.2020.0167 LK - <http://jq6am9xs3s.search.serialssolutions.com/?sid=EMBASE&issn=23983795&id=doi:10.3399%2FBJGPO.2020.0167&atitle=Antibiotic+prescribing+in+UK+out-of-hours+primary+care+services%3A+a+realist-informed+scoping+review+of+training>+

50. Aghlmandi S, Halbeisen FS, Saccilotto R, et al. Effect of Antibiotic Prescription Audit and Feedback on Antibiotic Prescribing in Primary Care: A Randomized Clinical Trial. *JAMA Internal Medicine* 2023;183(3):213-20. doi: 10.1001/jamainternmed.2022.6529 LK - <http://jq6am9xs3s.search.serialssolutions.com/?sid=EMBASE&issn=21686114&id=doi:10.1001%2Fjamainternmed.2022.6529&atitle=Effect+of+Antibiotic+Prescription+Audit+and+Feedback+on+Antibiotic+Prescribing+in+Primary+Care%3A>

51. Watier L, Cavalié P, Coignard B, et al. Comparing antibiotic consumption between two European countries: are packages an adequate surrogate for prescriptions? *Euro surveillance : bulletin Europeen sur les maladies transmissibles = European communicable disease bulletin* 2017;22(46) doi: 10.2807/1560-7917.ES.2017.22.46.17-00352

52. Atkins L, Chadborn T, Bondaronek P, et al. Content and mechanism of action of national antimicrobial stewardship interventions on management of respiratory tract infections in primary and community care. *Antibiotics* 2020;9(8):1-36. doi: <https://dx.doi.org/10.3390/antibiotics9080512> PT - Journal Article

53. Poss-Doering R, Kronsteiner D, Kamradt M, et al. Antibiotic prescribing for acute, non-complicated infections in primary care in Germany: baseline assessment in the cluster randomized trial ARena. *BMC Infectious Diseases* 2021;21(1):877-77. doi: 10.1186/s12879-021-06571-0

54. Palin V, Molter A, Belmonte M, et al. Antibiotic prescribing for common infections in UK general practice: variability and drivers. *The Journal of antimicrobial chemotherapy* 2019;74(8):2440-50. doi: <https://dx.doi.org/10.1093/jac/dkz163> PT - Journal Article, Observational Study, Research Support, Non-U.S. Gov't

55. Sijbom M, Buchner FL, Saadah NH, et al. Determinants of inappropriate antibiotic prescription in primary care in developed countries with general practitioners as gatekeepers: A systematic review and construction of a framework. *BMJ Open* 2023;13(5):e065006-e06. doi: 10.1136/bmjopen-2022-065006

56. Hummers-Pradier E, Ohse AM, Koch M, et al. Management of urinary tract infections in female general practice patients. *Fam Pract* 2005;22(1):71-7. doi: 10.1093/fampra/cmh720 [published Online First: 20050107]

57. Allison R, Lecky D, Beech E, et al. Local implementation of national guidance on management of common infections in primary care in England. *Pharmaceutical Journal* 2020 doi: 10.1211/pj.2020.20207599

58. Muijrers PE, Janknegt R, Sijbrandij J, et al. Prescribing indicators. Development and validation of guideline-based prescribing indicators as an instrument to measure the variation in the prescribing behaviour of general practitioners. *Eur J Clin Pharmacol* 2004;60(10):739-46. doi: 10.1007/s00228-004-0821-5 [published Online First: 20041026]

59. Bennett Institute for Applied Data Science, University of Oxford. OpenPrescribing.net 2024 [Available from: <https://openprescribing.net/> accessed December 2023.

60. Degnan KO, Cluzet V, David MZ, et al. Development and validation of antibiotic stewardship metrics for outpatient respiratory tract diagnoses and association of provider characteristics with inappropriate prescribing. *Infect Control Hosp Epidemiol* 2022;43(1):56-63. doi: 10.1017/ice.2021.44 [published Online First: 20210602]

61. Kern WV. Organization of antibiotic stewardship in Europe: the way to go. *Wien Med Wochenschr* 2021;171(Suppl 1):4-8. doi: 10.1007/s10354-020-00796-5 [published Online First: 20210209]

62. Kopsidas I, Vergnano S, Spyridis N, et al. A Survey on National Pediatric Antibiotic Stewardship Programs, Networks and Guidelines in 23 European Countries. *Pediatr Infect Dis J* 2020;39(11):e359-e62. doi: 10.1097/INF.0000000000002835

63. Buehrle DJ, Shively NR, Wagener MM, et al. Sustained Reductions in Overall and Unnecessary Antibiotic Prescribing at Primary Care Clinics in a Veterans Affairs Healthcare System Following a Multifaceted Stewardship Intervention. *Clin Infect Dis* 2020;71(8):e316-e22. doi: 10.1093/cid/ciz1180

64. Schuts EC, van den Bosch CM, Gyssens IC, et al. Adoption of a national antimicrobial guide (SWAB-ID) in the Netherlands. *Eur J Clin Pharmacol* 2016;72(2):249-52. doi: 10.1007/s00228-015-1969-x [published Online First: 20151022]

65. Fernandez-Urrusuno R, Flores-Dorado M, Moreno-Campoy E, Montero-Balosa MC. Selection of indicators for continuous monitoring of the impact of programs optimizing antimicrobial use in Primary Care. *Enferm Infecc Microbiol Clin* 2015;33(5):311-9. doi: 10.1016/j.eimc.2014.07.011 [published Online First: 20141022]

66. Rojo-Martín M, Peñalva G, Pinto C, et al. The PIRASOA programme: design, structure, organisation and indicators of a comprehensive regional Institutional Programme for the Prevention and Control of Healthcare-associated Infections and Antimicrobial Stewardship for hospitals and primary care settings in Andalusia, Spain V.2. *Preprint Protocolsio* 2018 doi: 10.17504/protocols.io.r3bd8in

67. Fernandez Urrusuno R, Montero Balosa MC, Perez Perez P, Pascual de la Pisa B. Compliance with quality prescribing indicators in terms of their relationship to financial incentives. *Eur J Clin Pharmacol* 2013;69(10):1845-53. doi: 10.1007/s00228-013-1542-4 [published Online First: 20130607]

68. Sanchez GV, Fleming-Dutra KE, Roberts RM, Hicks LA. Core Elements of Outpatient Antibiotic Stewardship. *MMWR Recomm Rep* 2016;65(6):1-12. doi: 10.15585/mmwr.rr6506a1 [published Online First: 20161111]

69. World Health Organization. Global action plan on antimicrobial resistance, 2015.

70. Busse R, Klazinga N, Panteli D, Quentin W. European Observatory Health Policy Series. Improving healthcare quality in Europe: Characteristics, effectiveness and implementation of different strategies. Copenhagen (Denmark): European Observatory on Health Systems and Policies

© World Health Organization (acting as the host organization for, and secretariat of, the European Observatory on Health Systems and Policies) and OECD (2019). 2019.

71. Health Prevention. PNCAR—National action plan on antimicrobial resistance 2017 2020 Italy, 2017.

72. Zoorob R, Sidani MA, Fremont RD, Kihlberg C. Antibiotic use in acute upper respiratory tract infections. *Am Fam Physician* 2012;86(9):817-22. [published Online First: 2012/11/02]

73. van den Broek d'Obrenan J, Verheij TJ, Numans ME, van der Velden AW. Antibiotic use in Dutch primary care: relation between diagnosis, consultation and treatment. *J Antimicrob Chemother* 2014;69(6):1701-7. doi: 10.1093/jac/dku005 [published Online First: 20140206]

74. O’Connor N, Breen R, Carton M, et al. Improving the quality of antibiotic prescribing through an educational intervention delivered through the out-of-hours general practice service in Ireland. *European Journal of General Practice* 2020;26(1):119-25. doi: 10.1080/13814788.2020.1784137

75. European Centre for Disease Prevention and Control. Antimicrobial consumption in the EU/EEA (ESAC-Net) Annual Epidemiological Report for 2021, 2022.

76. European Centre for Disease Prevention and Control. Surveillance of antimicrobial

consumption in Europe - report 2012, 2014.

77. DePestel DD, Eiland EH, 3rd, Lusardi K, et al. Assessing appropriateness of antimicrobial therapy: in the eye of the interpreter. *Clin Infect Dis* 2014;59 Suppl 3:S154-61. doi: 10.1093/cid/ciu548

78. van den Bosch CM, Geerlings SE, Natsch S, et al. Quality indicators to measure appropriate antibiotic use in hospitalized adults. *Clin Infect Dis* 2015;60(2):281-91. doi: 10.1093/cid/ciu747 [published Online First: 20140928]

79. Monnier AA, Schouten J, Le Marechal M, et al. Quality indicators for responsible antibiotic use in the inpatient setting: a systematic review followed by an international multidisciplinary consensus procedure. *J Antimicrob Chemother* 2018;73(suppl_6):vi30-vi39. doi: 10.1093/jac/dky116

80. Goossens H, Guillemot D, Ferech M, et al. National campaigns to improve antibiotic use. *Eur J Clin Pharmacol* 2006;62(5):373-9. doi: 10.1007/s00228-005-0094-7 [published Online First: 20060328]

81. ECDC EFSA Panel on Biological Hazards (BIOHAZ), EMA Committee for Medicinal Products for Veterinary Use (CVMP). ECDC, EFSA and EMA Joint Scientific Opinion on a list of outcome indicators as regards surveillance of antimicrobial resistance and antimicrobial consumption in humans and food-producing animals. *EFSA J* 2017;15(10):e05017. doi: 10.2903/j.efsa.2017.5017 [published Online First: 20171026]

82. Charra F, Berthelot P, Bergheau F. Impact of amoxicillin and oral amoxicillin-clavulanic acid defined daily doses on consumption indicators. *Med Mal Infect* 2019;49(3):208-13. doi: 10.1016/j.medmal.2018.10.009 [published Online First: 20181120]

83. Glinz D, Leon Reyes S, Saccilotto R, et al. Quality of antibiotic prescribing of Swiss primary care physicians with high prescription rates: a nationwide survey. *J Antimicrob Chemother* 2017;72(11):3205-12. doi: 10.1093/jac/dkx278

84. Ivanovska V, Hek K, Mantel Teeuwisse AK, et al. Antibiotic prescribing for children in primary care and adherence to treatment guidelines. *J Antimicrob Chemother* 2016;71(6):1707-14. doi: 10.1093/jac/dkw030 [published Online First: 20160305]

85. Balinskaite V, Johnson AP, Holmes A, Aylin P. The Impact of a National Antimicrobial Stewardship Program on Antibiotic Prescribing in Primary Care: An Interrupted Time Series Analysis. *Clinical Infectious Diseases* 2018;69(2):227-32. doi: 10.1093/cid/ciy902

86. Coenen S, Gielen B, Blommaert A, et al. Appropriate international measures for outpatient antibiotic prescribing and consumption: recommendations from a national data comparison of different measures. *J Antimicrob Chemother* 2014;69(2):529-34. doi: 10.1093/jac/dkt385 [published Online First: 20131001]

87. Sabuncu E, David J, Bernede-Bauduin C, et al. Significant reduction of antibiotic use in the community after a nationwide campaign in France, 2002-2007. *PLoS Med* 2009;6(6):e1000084. doi: 10.1371/journal.pmed.1000084 [published Online First: 20090602]

88. Sanchez GV, Fleming-Dutra KE, Roberts RM, Hicks LA. The Core Elements of Outpatient Antibiotic Stewardship: MMWR Recomm Rep, 2016.

89. Ashiru-Oredope D, Hopkins S, English Surveillance Programme for Antimicrobial U, Resistance Oversight G. Antimicrobial stewardship: English Surveillance Programme for Antimicrobial Utilization and Resistance (ESPAUR). *J Antimicrob Chemother* 2013;68(11):2421-3. doi: 10.1093/jac/dkt363 [published Online First: 20130911]

90. McNulty CA. Optimising antibiotic prescribing in primary care. *Int J Antimicrob Agents* 2001;18(4):329-33. doi: 10.1016/s0924-8579(01)00412-5

91. Ashiru-Oredope D, Sharland M, Charani E, et al. Improving the quality of antibiotic prescribing in the NHS by developing a new Antimicrobial Stewardship Programme: Start Smart--Then Focus. *J Antimicrob Chemother* 2012;67 Suppl 1:i51-63. doi: 10.1093/jac/dks202

92. Llor C, Bjerrum L. Antimicrobial resistance: risk associated with antibiotic overuse and initiatives to reduce the problem. *Ther Adv Drug Saf* 2014;5(6):229-41. doi: 10.1177/2042098614554919

93. UK Health Security Agency. English surveillance programme for antimicrobial utilisation and resistance (ESPAUR) Report 2021 to 2022: London: UK Health Security Agency, 2022.

94. Department of Health and Social Care. Contained and controlled The UK’s 20-year vision for antimicrobial resistance, 2019.

95. Williams MR, Greene G, Naik G, et al. Antibiotic prescribing quality for children in primary care: an observational study. *Br J Gen Pract* 2018;68(667):e90-e96. doi: 10.3399/bjgp18X694409 [published Online First: 20180115]

96. Hedin K, Strandberg EL, Grondal H, et al. Management of patients with sore throats in relation to guidelines: an interview study in Sweden. *Scand J Prim Health Care* 2014;32(4):193-9. doi: 10.3109/02813432.2014.972046 [published Online First: 20141103]

97. Jim ON. Tackling drug-resistant infections globally: final report and recommendations. 2016 doi: <https://doi.org/APO-63983>

98. Smith DRM, Dolk FCK, Pouwels KB, et al. Defining the appropriateness and inappropriateness of antibiotic prescribing in primary care. *J Antimicrob Chemother* 2018;73(suppl_2):ii11-ii18. doi: 10.1093/jac/dkx503

99. Bateman DN, Eccles M, Campbell M, et al. Setting standards of prescribing performance in primary care: use of a consensus group of general practitioners and application of standards to practices in the north of England. *Br J Gen Pract* 1996;46(402):20-5.

100. Johnson AP. Surveillance of antibiotic resistance. *Philos Trans R Soc Lond B Biol Sci* 2015;370(1670):20140080. doi: 10.1098/rstb.2014.0080

101. Kronman MP, Gerber JS, Grundmeier RW, et al. Reducing Antibiotic Prescribing in Primary Care for Respiratory Illness. *Pediatrics* 2020;146(3) doi: 10.1542/peds.2020-0038 [published Online First: 20200803]

102. Santos NSD, Marengo LL, Moraes FDS, Barberato Filho S. Interventions to reduce the prescription of inappropriate medicines in older patients. *Rev Saude Publica* 2019;53:7. doi: 10.11606/S1518-8787.2019053000781 [published Online First: 20190131]

103. Nathwani D, Sneddon J, Patton A, Malcolm W. Antimicrobial stewardship in Scotland: impact of a national programme. *Antimicrob Resist Infect Control* 2012;1(1):7. doi: 10.1186/2047-2994-1-7 [published Online First: 20120203]

104. Hawker JI, Smith S, Smith GE, et al. Trends in antibiotic prescribing in primary care for clinical syndromes subject to national recommendations to reduce antibiotic resistance, UK 1995-2011: analysis of a large database of primary care consultations. *J Antimicrob Chemother* 2014;69(12):3423-30. doi: 10.1093/jac/dku291 [published Online First: 20140804]

105. Rose J, Crosbie M, Stewart A. A qualitative literature review exploring the drivers influencing antibiotic over-prescribing by GPs in primary care and recommendations to reduce unnecessary prescribing. *Perspect Public Health* 2021;141(1):19-27. doi: 10.1177/1757913919879183 [published Online First: 20191021]

106. Malmros K, Huttner BD, McNulty C, et al. Comparison of antibiotic treatment guidelines for urinary tract infections in 15 European countries: Results of an online survey. *Int J Antimicrob Agents* 2019;54(4):478-86. doi: 10.1016/j.ijantimicag.2019.06.015 [published Online First: 20190620]

107. Fernandez R, Flores M, Vilches A, et al. Improving the appropriateness of antimicrobial use in primary care after implementation of a local antimicrobial guide in both levels of care. *Eur J Clin Pharmacol* 2014;70(8):1011-20. doi: 10.1007/s00228-014-1704-z [published Online First: 20140603]

108. Ashworth M, Golding S, Majeed A. Prescribing indicators and their use by primary care groups to influence prescribing. *J Clin Pharm Ther* 2002;27(3):197-204. doi: 10.1046/j.1365-2710.2002.00405.x

109. Ashworth M, Lea R, Gray H, et al. How are primary care organizations using financial incentives to influence prescribing? *J Public Health (Oxf)* 2004;26(1):48-51. doi: 10.1093/pubmed/fdh100
