# Supplementary material for: Setting targets for antibiotic use in general practice in Europe: A scoping review
Source: Eur J Gen Pract. 2024 Nov 28;30(1):2430507. doi: 10.1080/13814788.2024.2430507 (PMC11610282; doi:10.1080/13814788.2024.2430507)
Supplement: Supplemental Material [file IGEN_A_2430507_SM3297.zip › ejgp-2024-0174-File007.docx]

# Supplementary material 5. The characteristics of the included reports

| **n** | **ID record** | **Title** | **Objective of paper (if applicable)** | **Setting** | **Year (s) of the analysis** | **Country** | **National target** | **General practice target (local target)** | **Strategies to link national target to local area** | **Funding** | **Note** |
| --- | --- | --- | --- | --- | --- | --- | --- | --- | --- | --- | --- |
| ^1^ | D'Atri 2019 | Targets for the reduction of antibiotic use in humans in the Transatlantic Taskforce on Antimicrobial Resistance (TATFAR) partner countries | A questionnaire was developed by the European Centre for Disease Prevention and Control (ECDC) with input from TATFAR partners. The questionnaire comprised 12 questions and addressed whether the country had established or was planning to establish targets to reduce antibiotic use, the rationale, baseline and timeline for achieving the targets, how progress was monitored and the status of the targets | Ambulatory care (AC) | 2017 | Austria, Belgium, Bulgaria, Croatia, Czech Republic, Denmark, Estonia, Finland, France, Germany, Greece, Hungary, Iceland, Ireland, Italy, Latvia, Lithuania, Luxembourg, Malta, the Netherlands, Norway, Poland, Romania, Slovakia, Slovenia, Spain, Sweden, the UK | Yes, for AC | No | Yes | Not mentioned | -Malta only reported hospital care targets.  -Denmark, Italy, Germany, Greece, Hungary, Iceland, Ireland, Latvia, Lithuania, Luxembourg, Poland, Romania, Slovakia, Spain, Austria, Bulgaria, Croatia, Czech Republic, Estonia and Finland had not established targets for the reduction of antimicrobial use in humans, TATFAR survey, 2017 |
| ^2^ | Howard 2017 | ESGAP inventory of target indicators assessing antibiotic prescriptions: A cross-sectional survey | To make an inventory of indicators that assess antibiotic prescriptions and are linked to specific targets and incentives, at a national level | Primary care / General practitioners | 2017 | Austria, Belgium, Bulgaria, Croatia, the Czech Republic, France, Germany, Greece, Ireland, Italy, Malta, the Netherlands, Norway, Portugal, Romania, Slovenia, Spain, Sweden, Switzerland, the UK | Yes | Yes | Yes | This study was carried out as part of our routine work | It did not mention targets for Netherland, Switzerland, Sweden, Austria, Czechia, Germany, Ireland, Italy, Malta, Romania and Slovenia  It only mentioned hospital setting targets for Croatia, Bulgaria, Greece and Portugal |
| ^3^ | Adriaenssens 2014 | Quality of antibiotic prescription during office hours and out-of-hours in Flemish primary care, using European quality indicators. | To assess the feasibility of calculating values for these 21 antibiotic prescribing quality indicators (APQI) using primary care databases; and to assess the quality of antibiotic prescribing in office hours and out-of-hours general practice | Office hours and out-of-hours general practice | 2003 - 2010 | Belgium | No | They did not mention targets. However, they used a range of acceptable values as a benchmark proposed by the ESAC project | No | Not mentioned |  |
| ^4^ | Bruyndonckx 2021 | Antibiotic use and resistance in Belgium: the impact of two decades of multi-faceted campaigning | To present an overview of almost two decades of multi-faceted campaigning by the Belgian Antibiotic Policy Coordination Committee (BAPCOC) and partners, and its impact on public and prescribers’ awareness, outpatient antibiotic use, its cost and antimicrobial resistance in Belgium | Primary care | 1987 - 2017 | Belgium | Yes, primary care | No | Yes | Research Foundation – Flanders (FWO) (BE) [12I6319N] |  |
| ^5^ | Colliers 2019 | Antibiotic prescribing quality in out-of-hours primary care and critical appraisal of disease-specific quality indicators | To assess the quality of antibiotic prescribing in OOH primary care to inform the implementation of future interventions to improve the antibiotic prescribing quality in this specific setting . More specifically, we aim to describe antibiotic prescribing in Belgian OOH primary care by indication, assess its quality by updating values for ESAC’s disease-specific APQI and critically appraise these APQI | Primary care | 2016 - 2018 | Belgium | No | They did not mention targets. However, they used a range of acceptable values as a benchmark proposed by the ESAC project | No | The Faculty of Medicine and Health Sciences of the University of Antwerp. The iCAREdata database is funded by the Research Foundation Flanders |  |
| ^6^ | Vellinga 2023 | Disease-Specific Quality Indicators for Outpatient Antibiotic Prescribing for Respiratory Infections (ESAC Quality Indicators) Applied to Point Prevalence Audit Surveys in General Practices in 13 European Countries | The collected data provided the opportunity to compare and evaluate the antibiotic prescribing quality using ESAC QI between and within participating general practices in 13 European countries and identify opportunities for antibiotic stewardship | General practice | 2022 | Belgium, Croatia, Denmark, Germany, Greece, Ireland, Poland, Romania, Spain, the UK | No | They did not mention targets. However, they used a range of acceptable values as a benchmark proposed by the ESAC project | No | The Innovative Medicine Initiative 2 Joint Undertaking, grant number 820755 (VALUE-Dx) | - |
| ^7^ | Tyrstrup 2017 | Antibiotic prescribing in relation to diagnoses and consultation rates in Belgium, the Netherlands and Sweden: use of European quality indicators | To assess the quality of antibiotic prescribing in primary care in Belgium, the Netherlands and Sweden using European disease-specific antibiotic prescribing quality indicators (APQI) and taking into account the threshold to consult and national guidelines | General practice | 2012 | Belgium, the Netherlands, Sweden | No | They did not mention targets. However, they used a range of acceptable values as a benchmark proposed by the European Surveillance of Antimicrobial Consumption project (ESAC) | No | Not mentioned | - |
| ^8^ | Vojvodić 2022 | How effective is a brief educational intervention on prescribing first-line antibiotics in acute cystitis? A quasi-experimental study among general practitioners in Croatia | To evaluate the effectiveness of a brief educational intervention among general practitioners (GPs) in Croatia targeted at increasing the prescription of first-line antibiotics in uncomplicated cystitis and reducing the use of fluoroquinolones. A secondary aim was to assess the quality of prescribing according to the European Surveillance of Antimicrobial Consumption (ESAC) quality indicators for outpatient antimicrobial utilization | General practice | 2019 | Croatia | No | They did not mention targets. However, they used a range of acceptable values as a benchmark proposed by the ESAC project | No | None |  |
| ^9^ | Saust 2018 | Quality assessment in general practice: diagnosis and antibiotic treatment of acute respiratory tract infections. | To investigate areas in need of quality improvement within the diagnostic process and antibiotic treatment of acute respiratory tract infections (RTIs) in Danish general practice by using quality indicators (QIs). | General practice | 2017 | Denmark | No | They did not mention targets. However, they used a range of acceptable values as a benchmark proposed by the ESAC project | No | Audit Project Odense |  |
| ^10^ | Michalsen 2023 | Regional and national antimicrobial stewardship activities: a survey from the Joint Programming Initiative on Antimicrobial Resistance - Primary Care Antibiotic Audit and Feedback Network (JPIAMR-PAAN) | An overview of existing and planned A&F initiatives as part of AMS programmes in primary care within JPIAMR-PAAN | Primary care | The survey was available from 21 October 2021 to 15 January 2022 | Denmark, France, Italy, the Netherlands, Norway, Spain, Sweden, Switzerland, the United Kingdom (UK) | No | No | Yes | The Joint Programming Initiative on Antimicrobial Resistance (JPIAMR) (Canadian Institute of Health Research grant number 448378) | Switzerland: not relevant information |
| ^11^ | Thilly 2020 | Proxy indicators (PIs) to estimate appropriateness of antibiotic prescriptions by general practitioners: a proof-of-concept cross-sectional study based on reimbursement data, north-eastern France 2017 | To: (i) define PIs for appropriateness of antibiotic use based on these routine data; and (ii) assess the PIs’ performance scores and their clinimetric properties using a large regional reimbursement database | General practice | 2017 | France | No | Yes (research-focused) | No | Not mentioned |  |
| ^12^ | Simon 2022 | Factors associated with the appropriateness of antibiotics prescribed in French general practice: a cross-sectional study using reimbursement databases | To identify (i) clusters of GPs according to their appropriateness score based on these proxy indicators results, and (ii) GPs’, patients’ and practices’ characteristics associated with inappropriate prescriptions | General practice | 2019 | France | No | Yes (research-focused) | No | This research did not receive any specific grant from funding agencies in the public, commercial, or not-for-profit sectors |  |
| ^13^ | Simon 2024 | Selection of proxy indicators estimating the appropriateness of antibiotic prescriptions in general practice: a national consensus procedure in France | To select a set of PIs based on the 10 developed by Thilly et al. to estimate the appropriateness of antibiotic prescriptions by GPs, through a RAND-modified Delphi consensus procedure.  To evaluate the clinimetric properties (measurability, applicability and potential room for improvement) of the selected PIs. | General practice | July 2021 and January 2022 | France | No | Yes (research-focused) | No | This work was carried out as part of our routine work |  |
| ^14^ | Arias 2024 | Identifying General Practitioners’ Antibiotic Prescribing  Profiles Based on National Health Reimbursement Data | To identify prescriber profiles using novel indicators for targeted antimicrobial stewardship programs (ASPs) in general practice. | General practice | 2018 | France | No | Yes (research-focused) | No | Not mentioned |  |
| ^15^ | Fernandez Urrusuno 2013 | Compliance with quality prescribing indicators in terms of their relationship to financial incentives | To develop quality prescribing indicators for general practitioners (GPs) who are non-monitored and not included in pay-for-performance programs, and to determine compliance with incentivized and non-incentivized indicators | General practice | 2007 | Spain - andalusia | No | Yes (research-focused) | No | The Spanish Society of Primary Care Pharmacists (SEFAP) |  |
| ^16^ | Molstad 2017 | Lessons learnt during 20 years of the swedish strategic programme against antibiotic resistance | To describe the main strategies of the programme: committed work at the local and national levels; monitoring of antibiotic use for informed decision-making; a national target for antibiotic prescriptions; surveillance of antibiotic resistance for local, national and global action; tracking resistance trends; infection control to limit spread of resistance; and communication to raise awareness for action and behavioural change | Outpatient care | 1997 - 2017 | Sweden | Yes, for Outpatient care | No | Yes | Not mentioned | - |
| ^17^ | Public Health Agency of Sweden 2019 | Strama's goals for antibiotic use in outpatient care | Strama would like to propose a number of targets for the use of antibiotics in outpatient care. The aim is to strive to maintain the relatively favourable resistance situation that Sweden has for as long as possible. | Outpatient care | 2008 | Sweden | Yes, for Outpatient care | No | No | Not mentioned | - |
| ^18^ | Public Health Agency of Sweden 2014 | Swedish work against antibiotic resistance  Tools, working methods and experiences | The report has been prepared through interviews with experts with experience of antibiotic resistance work and is also based on previous reports from, among others, the former national Strama, the Public Health Agency - formerly the Institute for Infection Control, and the National Board of Health and Welfare | Outpatient care and General practice | 1994 - 2014 | Sweden | Yes, for Outpatient care | Yes | Yes | The project has been financed by The Swedish International Development Cooperation Agency | - |
| ^19^ | Swedres-Svarm 2020 | Sales of antibiotics and occurrence of antibiotic resistance in Sweden 2020 | A report on Swedish Antibiotic Sales and Resistance in Human Medicine (Swedres) and Swedish Veterinary Antibiotic Resistance Monitoring (Svarm) | Outpatient care | 2019 - 2020 | Sweden | Yes, Outpatient care | No | Yes | Not applicable | - |
| ^20^ | Plate 2020 | Treatment of urinary tract infections in Swiss primary care: quality and determinants of antibiotic prescribing | To determine the prescribing patterns and the quality of antibiotic prescribing in ambulatory UTI and report factors associated with appropriate or inappropriate antibiotic prescribing | General practice | 2017 - 2018 | Switzerland | No | They did not mention targets. However, they used a range of acceptable values as a benchmark proposed by the ESAC project | No | Grants from SwissLife, Blumenau-Léonie Hartmann Foundation, and Innova Foundation |  |
| ^21^ | McCloskey 2023 | Antibiotic prescribing trends in primary care 2014-2022 | To capture prescribing trends for commonly prescribed antibiotics in primary care in England with a specific focus on respiratory and urinary tract infections | General practice | Trend 2014 -2022 | The UK | Yes | No | No | LM was funded to work on this project by the Society for Applied Microbiology (now Applied Microbiology International) Summer Studentship | - |
| ^22^ | Niazi-Ali 2022 | Broad spectrum antibiotic stewardship by quality improvement methods | Broad spectrum antibiotic prescribing is often seen as a proxy marker of increasing resistance within a population and so it is important that they are used sparingly, to avoid drug-resistant bacteria developing | General practice | 2019 - 2020 financial year | The UK | Yes, for primary care | Yes | Yes | Not mentioned | - |
| ^23^ | Nathwani 2011 | Scottish Antimicrobial Prescribing Group (SAPG): development and impact of the Scottish National Antimicrobial Stewardship Programme | To explore the development and impact of the Scottish National Antimicrobial Stewardship Programme | Primary care | 2005 - 2009 | The UK - Scotland | Yes, for Primary care | Yes | Yes | The Scottish Antimicrobial Stewardship Programme is supported by funding from the Scottish Government Health Department | - |
| ^24^ | Nathwani 2012 | Antimicrobial stewardship in Scotland: impact of a national programme | Not applicable (short report) | Primary care | 2008 - 2011 | The UK - Scotland | Yes, for Primary care | Yes | No | Not mentioned | - |
| ^25^ | British Society for Antimicrobial Chemotherapy 2018 | Antimicrobial stewardship from principles to practice | To encourage a whole team approach it is beneficial to share data at multi-professional meetings and this can be done informally at ward meetings or via hospital or directorate level audit meetings or in a community setting with all staff in a clinic. | General practice | 2013 - 2016 | The UK - Scotland | No | Yes | Yes | Not mentioned |  |
| ^26^ | Antimicrobial Resistance and Healthcare Associated Infection Scotland 2023 | Scottish One Health Antimicrobial Use and Antimicrobial Resistance in 2022 | Not applicable | Primary care | 2019 - 2024 | The UK - Scotland | Yes | No | No | Not applicable |  |
| ^27^ | Allison 2020 | What antimicrobial stewardship strategies do NHS commissioning organizations implement in primary care in England? | To identify and explore strategies that English NHS commissioning organizations implemented to improve antimicrobial stewardship (AMS) within primary care | Primary care | 2017 | The UK -England | No | No | Yes | Public Health England (PHE) | - |
| ^28^ | Johnson 2017 | Improving feedback of surveillance data on antimicrobial consumption, resistance and stewardship in England: Putting the data at your Fingertips | The provision of better access to and use of surveillance data is a key component of the UK 5 Year Antimicrobial Resistance (AMR) Strategy. Since April 2016, PHE has made data on practice (infection prevention and control; antimicrobial stewardship) and outcome (prevalence of AMR, antibiotic use and healthcare-associated infections) available through Fingertips | Level of National Health Service (NHS) acute trusts, Clinical Commissioning Groups or general practitioner practices | 2016 | The UK -England | No | No | Yes | All work was performed as part of the routine work of the contributing authors. | - |
| ^29^ | Anyanwu 2020 | Conceptualising the Integration of Strategies by Clinical Commissioning Groups in England towards the Antibiotic Prescribing Targets for the Quality Premium Financial Incentive Scheme: A Short Report. | This paper briefly reports the outcome of a workshop exploring the experiences of antimicrobial stewardship (AMS) leads within Clinical Commissioning Groups in selecting and adopting strategies to help achieve the QP antibiotic targets. | Primary care | 2019 | The UK -England | No | No | Yes | The Economic and Social Research Council (grant: ES/P008232/1) | - |
| ^30^ | Gold 2022 | Using text and charts to provide social norm feedback to general practices with high overall and high broad-spectrum antibiotic prescribing: a series of national randomised controlled trials | (1) The impact on broad-spectrum prescribing of sending a social norm feedback letter targeting broad-spectrum prescribing compared to sending a social norm feedback letter targeting overall antibiotic prescribing (2) The impact on broad-spectrum prescribing of sending a social norm feedback letter targeting broad-spectrum prescribing compared to sending no letter (3) The impact on overall prescribing of adding a bar chart to a social norm feedback letter targeting overall prescribing that was text-only | General practice | 2017 - 2019 | The UK -England | Yes, for primary care | Yes | Yes | PHE |  |
| ^31^ | Anyanwu 2020a | Investigating the mechanism of impact and differential effect of the Quality Premium scheme on antibiotic prescribing in England: A longitudinal study | To investigate whether the effects of the Quality Premium (QP), which provided performance-related financial incentives to clinical commissioning groups, could be explained by practice characteristics that contribute to variations in antibiotic prescribing | General practice | 2014 - 2016 | The UK -England | Yes, for primary care | Yes | No | The Economic and Social Research Council (grant: ES/P008232/1) |  |
| ^32^ | Pouwels 2018 | Actual versus ‘ideal’ antibiotic prescribing for common conditions in English primary care | Previous work based on guidelines and expert opinion identified ‘ideal’ prescribing proportions—the overall proportion of consultations that should result in an antibiotic prescription—for common infectious conditions. Here, actual condition-specific prescribing proportions in primary care in England were compared with ideal prescribing proportions identified by experts. | General practice | 2013 - 2015 | The UK -England | Yes | Yes (research-focused) | No | PHE |  |
| ^33^ | Smieszek 2018 | Potential for reducing inappropriate antibiotic prescribing in English primary care | To identify and quantify inappropriate systemic antibiotic prescribing in primary care in England, and ultimately to determine the potential for reduction in prescribing of antibiotics. | General practice | 2013 - 2015 | The UK -England | No | Yes (research-focused) | No | PHE | - |
| ^34^ | NHS England and NHS Improvement 2024 | Webkits -Antimicrobial stewardship | Not applicable | Integrated care boards, health boards, locations, old boundaries, PCN/cluster and Practice | 2023 - 2024 | The UK -England | Yes | Yes | Yes | Not applicable |  |
| ^35^ | UK Health Security Agency 2023 | English surveillance programme for antimicrobial utilisation and resistance (ESPAUR) Report 2022 to 2023 | Not applicable | Primary care | 2022 - 2023 | The UK -England | Yes | Yes | Yes | Not applicable |  |
| ^36^ | Knowles 2024 | A systematic review of national interventions and policies to optimize antibiotic use in healthcare settings in England | To identify and assess the effectiveness of national antibiotic optimization interventions in primary  and secondary care in England (2013–2022) | Primary care | 2013 to 2022 | The UK -England | Yes, for primary care | No | Yes | The Economic Social Research Council (ESRC) (grant number ES/P000592/1) |  |

**National actions Plans on Antimicrobial Resistance**

| **n** | **Country** | **Title** | **Period** | **National target** | **General practice target (local target)** | **Strategies to link national target to local area** | **Comments** |
| --- | --- | --- | --- | --- | --- | --- | --- |
| ^37 38^ | Austria | National Action Plan on Antibiotic Resistance | Not clear, published in 2021 | No | No | No | Not mentioned targets  Not found an update NAP |
|  |  | TrACSS report | 2023 | - | - | - | TrACSS report, Austria has developed and implemented NAP AMR. Also, it has a monitoring and evaluation plan for the NAP AMR |
| ^39^ | Belgium | “One Health” National Action Plan on the Fight Against Antimicrobial Resistance (AMR) | 2020 - 2024 | Yes, outpatient practice | No | No | None |
| ^40^ | Bulgaria | TrACSS report | 2023 | - | - | - | Not found NAP.  TrACSS report, Bulgaria has developed NAP AMR, however, it has not been implementing NAP AMR. Also, it does not have a monitoring and evaluation plan for the NAP AMR |
| ^41 42^ | Croatia | National control program resistance of bacteria to antibiotics | 2017 - 2021 | No | No | No | Not mentioned the target  Not found an update NAP |
|  |  | TrACSS report | 2023 | - | - | - | TrACSS report, Croatia has developed and implemented NAP AMR. Also, it has a monitoring and evaluation plan for the NAP AMR |
| ^43 44^ | Cyprus | National Strategy for Addressing it Microbial Resistance to Antibiotics | Not clear, published in 2012 | No | No | No | Not mentioned targets  Not found an update NAP |
|  |  | TrACSS report | 2023 | - | - | - | TrACSS report, Cyprus has developed NAP AMR, however, country has not been implementing NAP AMR. Also, it does not have a monitoring and evaluation plan for the NAP AMR |
| ^45^ | Czech Republic | TrACSS report | 2023 | - | - | - | Not found NAP  TrACSS report, Czech Republic has not developed NAP AMR. However, country in the process of revising the NAP AMR or developing a new one, and has a monitoring and evaluation plan for the NAP AMR |
| ^46^ | Denmark | National action plan on antibiotics in human health care - Three measurable goals for a reduction of antibiotic consumption towards 2020 | 2016 - 2020 | Yes, primary care | No | Yes | Not found an update NAP |
| ^47^ | Estonia | TrACSS report | 2023 | - | - | - | Not found NAP  TrACSS report, Estonia has not developed NAP AMR. However, country in the process of revising the NAP AMR or developing a new one |
| ^48 49^ | Finland | The National Action Plan on Antimicrobial Resistance | 2017–2021 | No | No | No | Not mentioned targets  Not found an update NAP |
|  |  | TrACSS report | 2023 | - | - | - | TrACSS report, Finland has developed and implemented NAP AMR. However, it does not have a monitoring and evaluation plan for the NAP AMR |
| ^50^ | France | National Strategy for Preventing Infections and Antibiotic Resistance | 2022 -2025 | Yes, community care | No | Yes | None |
| ^51 52^ | Germany | DART 2020 - Fighting antibiotic resistance for the good of both humans and animals | 2015 – 2020 | No | No | No | Not mentioned targets  Not found an update NAP |
|  |  | TrACSS report | 2023 | - | - | - | TrACSS report, Germany has developed and implemented NAP AMR. However, it does not have a monitoring and evaluation plan for the NAP AMR |
| ^53^ | Greece | National Action Plan for Combating Antimicrobial Resistance in Greece in the Framework of Unified Health | 2019 - 2023 | Yes, Community and out-hospital environment | No | No | Not found an update NAP |
| ^54 55^ | Hungary | National Action Plan on the infection control and antimicrobial resistance based on the “One Health” Approach, in unity with the veterinary medicinal products | Not clear, published in 2019 | No | No | No | Not mentioned targets  Not found an update NAP |
|  |  | TrACSS report | 2023 | - | - | - | TrACSS report, Hungary has developed but has not implemented NAP AMR. Also, it does not have a monitoring and evaluation plan for the NAP AMR |
| ^56 57^ | Iceland | Report of a working group on measures to reduce the spread of antibiotic-resistant bacteria in Iceland | Not clear, published in 2017 | No | No | No | Not mentioned targets  Not found an update NAP |
|  |  | TrACSS report | 2023 | - | - | - | TrACSS report, Iceland has developed but has not implemented NAP AMR. Also, it does not have a monitoring and evaluation plan for the NAP AMR |
| ^58^ | Ireland | Health Service Executive Antimicrobial Resistance Infection Control (AMRIC) action plan | 2022 - 2025 | Yes, Community | Yes | Yes | None |
| ^59^ | Italy | National action Plan to Antibiotic Resistance (PNCAR) | 2022 - 2025 | Yes, local area | No | No | None |
| ^60^ | Latvia | Limiting antimicrobial resistance and a prudent antibiotic use plan "One health" | 2023 - 2027 | No | No | No | Not mentioned targets |
| ^61^ | Lithuania | Action plan for the prevention and control of the spread of antimicrobial-resistant microorganisms and hospital-acquired infections | 2023 - 2027 | No | No | No | Not mentioned targets |
| ^62^ | Luxembourg | National Plan Antibiotics | 2018 - 2022 (extended until 2024) | No | No | No | Not clear targets. They mentioned an indicator but not target.  INDICATOR: Reduction in antibiotic consumption by class of antibiotics (macrolides, tetracyclines, quinolones, etc.)  SOURCES OF DATA: Report annual of the consumption antibiotics  FREQUENCY OF THE COLLECTION DATA: Annual  REFERENCE: 2017 |
| ^63^ | Malta | A Strategy and Action Plan for the Prevention and Containment of Antimicrobial Resistance in Malta | 2020 - 2028 | No | No | No | Not mentioned targets |
| ^64^ | Netherlands | Dutch national action plan on AMR | 2015 - 2019 | Yes, national | No | No | Not found an update NAP |
| ^65^ | Norway | Strategy National Strategy against Antibiotic Resistance | 2015 - 2020 | Yes, national | No | No | Not found an update NAP |
| ^66 67^ | Poland | National programme to protect antibiotics | 2016 - 2020 | No | No | No | Not mentioned targets  Not found an update NAP |
|  |  | TrACSS report | 2023 | - | - | - | TrACSS report, Poland has not developed and implemented NAP AMR. Also, it has a monitoring and evaluation plan for the NAP AMR |
| ^68^ | Portugal | National Plan to Combat Antimicrobial Resistance | 2019-2023 | Yes, community | No | No | Not found an update NAP |
| ^69^ | Romania | Action plan for the implementation of the Strategy for the prevention and limitation of healthcare-associated infections and combating the phenomenon of antimicrobial resistance | 2023 - 2030 | No | No | No | Not mentioned targets |
| ^70 71^ | Slovakia | National Action Plan on Antimicrobial Resistance in the Slovak Republic for the period | 2019 - 2021 | No | No | No | Not mentioned targets  Not found an update NAP |
|  |  | TrACSS report | 2023 | - | - | - | TrACSS report, Slovakia has developed and implemented NAP AMR. Also, it has a monitoring and evaluation plan for the NAP AMR |
| ^72^ | Slovenia | National strategy "one health" for the control of microbial resistance | 2019 - 2024 | Yes, Ambulatory care | No | No | None |
| ^73^ | Spain | National Plan against Antibiotic Resistance (PRAN) | 2022 -2024 | No | No | No | Not mentioned the target  There is currently a study underway of antibiotic consumption indicators in the community sector linked to indications or diagnoses |
| ^74^ | Sweden | Swedish Strategy to Combat Antibiotic Resistance | 2024 -2025 | Yes, Primary care | No | Yes | None |
| ^75 76^ | Switzerland | Swiss Antibiotic Resistance Report (SARR) 2022. Usage of Antibiotics and Occurrence of Antibiotic Resistance in Switzerland | 2022 | No | No | No | Not found NAP, SARR is the national report on the antibiotic resistance situation in Switzerland.  It does not mention a clear target.  They compared indicators for the consumption of antibiotics for systemic use in the outpatient setting with countries participating in the ESAC-Net and classified the value of indicator in better quality indicator (quartile) |
|  |  | TrACSS report | 2023 | No | No | No | TrACSS report, Switzerland has developed and implemented NAP AMR. Also, it has a monitoring and evaluation plan for the NAP AMR |
| ^77^ | The UK | Tackling antimicrobial resistance: The UK’s five-year national action plan | 2019 - 2024 | Yes, national | No | Yes | None |

**References**

1. D'Atri F, Arthur J, Blix HS, et al. Targets for the reduction of antibiotic use in humans in the Transatlantic Taskforce on Antimicrobial Resistance (TATFAR) partner countries. *Euro Surveill* 2019;24(28) doi: 10.2807/1560-7917.ES.2019.24.28.1800339

2. Howard P, Huttner B, Beovic B, et al. ESGAP inventory of target indicators assessing antibiotic prescriptions: a cross-sectional survey. *J Antimicrob Chemother* 2017;72(10):2910-14. doi: 10.1093/jac/dkx243

3. Adriaenssens N, Bartholomeeusen S, Ryckebosch P, Coenen S. Quality of antibiotic prescription during office hours and out-of-hours in Flemish primary care, using European quality indicators. *Eur J Gen Pract* 2014;20(2):114-20. doi: 10.3109/13814788.2013.828200 [published Online First: 20130902]

4. Bruyndonckx R, Coenen S, Hens N, et al. Antibiotic use and resistance in Belgium: the impact of two decades of multi-faceted campaigning. *Acta Clin Belg* 2021;76(4):280-88. doi: 10.1080/17843286.2020.1721135 [published Online First: 20200205]

5. Colliers A, Adriaenssens N, Anthierens S, et al. Antibiotic Prescribing Quality in Out-of-Hours Primary Care and Critical Appraisal of Disease-Specific Quality Indicators. *Antibiotics (Basel)* 2019;8(2) doi: 10.3390/antibiotics8020079 [published Online First: 20190612]

6. Vellinga A, Luke-Currier A, Garzon-Orjuela N, et al. Disease-Specific Quality Indicators for Outpatient Antibiotic Prescribing for Respiratory Infections (ESAC Quality Indicators) Applied to Point Prevalence Audit Surveys in General Practices in 13 European Countries. *Antibiotics (Basel)* 2023;12(3) doi: 10.3390/antibiotics12030572 [published Online First: 20230314]

7. Tyrstrup M, van der Velden A, Engstrom S, et al. Antibiotic prescribing in relation to diagnoses and consultation rates in Belgium, the Netherlands and Sweden: use of European quality indicators. *Scand J Prim Health Care* 2017;35(1):10-18. doi: 10.1080/02813432.2017.1288680 [published Online First: 20170303]

8. Vojvodić Ž, Mimica S. How effective is a brief educational intervention on prescribing first-line antibiotics in acute cystitis? A quasi-experimental study among general practitioners in Croatia. *Croatian Medical Journal* 2022;63(4):262-369. doi: 10.3325/cmj.2022.63.362

9. Saust LT, Bjerrum L, Siersma V, et al. Quality assessment in general practice: diagnosis and antibiotic treatment of acute respiratory tract infections. *Scand J Prim Health Care* 2018;36(4):372-79. doi: 10.1080/02813432.2018.1523996 [published Online First: 20181008]

10. Michalsen BO, Xu AXT, Alderson SL, et al. Regional and national antimicrobial stewardship activities: a survey from the Joint Programming Initiative on Antimicrobial Resistance—Primary Care Antibiotic Audit and Feedback Network (JPIAMR-PAAN). *JAC-Antimicrobial Resistance* 2023;5(2) doi: 10.1093/jacamr/dlad048

11. Thilly N, Pereira O, Schouten J, et al. Proxy indicators to estimate appropriateness of antibiotic prescriptions by general practitioners: a proof-of-concept cross-sectional study based on reimbursement data, north-eastern France 2017. *Euro Surveill* 2020;25(27) doi: 10.2807/1560-7917.ES.2020.25.27.1900468

12. Simon M, Thilly N, Pereira O, Pulcini C. Factors associated with the appropriateness of antibiotics prescribed in French general practice: a cross-sectional study using reimbursement databases. *Clin Microbiol Infect* 2022;28(4):609 e1-09 e6. doi: 10.1016/j.cmi.2021.08.026 [published Online First: 20210906]

13. Simon M, Bocquier A, Pereira O, et al. Selection of proxy indicators estimating the appropriateness of antibiotic prescriptions in general practice: a national consensus procedure in France. *JAC Antimicrob Resist* 2024;6(2):dlae059. doi: 10.1093/jacamr/dlae059 [published Online First: 20240416]

14. Arias P, Matta M, Strazzulla A, et al. Identifying General Practitioners' Antibiotic Prescribing Profiles Based on National Health Reimbursement Data. *Open Forum Infect Dis* 2024;11(4):ofae172. doi: 10.1093/ofid/ofae172 [published Online First: 20240321]

15. Fernandez Urrusuno R, Montero Balosa MC, Perez Perez P, Pascual de la Pisa B. Compliance with quality prescribing indicators in terms of their relationship to financial incentives. *Eur J Clin Pharmacol* 2013;69(10):1845-53. doi: 10.1007/s00228-013-1542-4 [published Online First: 20130607]

16. Molstad S, Lofmark S, Carlin K, et al. Lessons learnt during 20 years of the Swedish strategic programme against antibiotic resistance. *Bull World Health Organ* 2017;95(11):764-73. doi: 10.2471/BLT.16.184374 [published Online First: 20171003]

17. Public Health Agency of Sweden, STRAMA. Strama's goals for antibiotic use in outpatient care 2019 [Available from: <https://strama.se/strategiska-dokument/>.

18. Public Health Agency of Sweden. Swedish work against antibiotic resistance Tools, working methods and experiences 2014 [Available from: <https://strama.se/strategiska-dokument/>.

19. Swedres-Svarm. Sales of antibiotics and occurrence of antibiotic resistance in Sweden: Solna/Uppsala, 2020.

20. Plate A, Kronenberg A, Risch M, et al. Treatment of urinary tract infections in Swiss primary care: quality and determinants of antibiotic prescribing. *BMC Fam Pract* 2020;21(1):125. doi: 10.1186/s12875-020-01201-1 [published Online First: 20200701]

21. McCloskey AP, Malabar L, McCabe PG, et al. Antibiotic prescribing trends in primary care 2014-2022. *Res Social Adm Pharm* 2023;19(8):1193-201. doi: 10.1016/j.sapharm.2023.05.001 [published Online First: 20230508]

22. Niazi-Ali S, Bircher J. Broad spectrum antibiotic stewardship by quality improvement methods. *Int J Risk Saf Med* 2022;33(S1):S35-S40. doi: 10.3233/JRS-227021

23. Nathwani D, Sneddon J, Malcolm W, et al. Scottish Antimicrobial Prescribing Group (SAPG): development and impact of the Scottish National Antimicrobial Stewardship Programme. *Int J Antimicrob Agents* 2011;38(1):16-26. doi: 10.1016/j.ijantimicag.2011.02.005 [published Online First: 20110422]

24. Nathwani D, Sneddon J, Patton A, Malcolm W. Antimicrobial stewardship in Scotland: impact of a national programme. *Antimicrob Resist Infect Control* 2012;1(1):7. doi: 10.1186/2047-2994-1-7 [published Online First: 20120203]

25. British Society for Antimicrobial Chemotherapy. Antimicrobial Stewardship: From Principles to Practice2018.

26. Antimicrobial Resistance and Healthcare Associated Infection Scotland. Scottish One Health Antimicrobial Use and Antimicrobial Resistance in 2022, 2023.

27. Allison R, Lecky DM, Beech E, et al. What antimicrobial stewardship strategies do NHS commissioning organizations implement in primary care in England? *JAC Antimicrob Resist* 2020;2(2):dlaa020. doi: 10.1093/jacamr/dlaa020 [published Online First: 20200514]

28. Johnson AP, Muller-Pebody B, Budd E, et al. Improving feedback of surveillance data on antimicrobial consumption, resistance and stewardship in England: putting the data at your Fingertips. *J Antimicrob Chemother* 2017;72(4):953-56. doi: 10.1093/jac/dkw536

29. Anyanwu PE, Borek AJ, Tonkin-Crine S, et al. Conceptualising the Integration of Strategies by Clinical Commissioning Groups in England towards the Antibiotic Prescribing Targets for the Quality Premium Financial Incentive Scheme: A Short Report. *Antibiotics (Basel)* 2020;9(2) doi: 10.3390/antibiotics9020044 [published Online First: 20200123]

30. Gold N, Sallis A, Saei A, et al. Using text and charts to provide social norm feedback to general practices with high overall and high broad-spectrum antibiotic prescribing: a series of national randomised controlled trials. *Trials* 2022;23(1):511. doi: 10.1186/s13063-022-06373-y [published Online First: 20220618]

31. Anyanwu PE, Pouwels K, Walker A, et al. Investigating the mechanism of impact and differential effect of the Quality Premium scheme on antibiotic prescribing in England: a longitudinal study. *BJGP Open* 2020;4(3) doi: 10.3399/bjgpopen20X101052 [published Online First: 20200825]

32. Pouwels KB, Dolk FCK, Smith DRM, et al. Actual versus 'ideal' antibiotic prescribing for common conditions in English primary care. *J Antimicrob Chemother* 2018;73(suppl_2):19-26. doi: 10.1093/jac/dkx502

33. Smieszek T, Pouwels KB, Dolk FCK, et al. Potential for reducing inappropriate antibiotic prescribing in English primary care. *J Antimicrob Chemother* 2018;73(suppl_2):ii36-ii43. doi: 10.1093/jac/dkx500

34. NHS England and NHS Improvement. Webkits -Antimicrobial stewardship 2024 [Available from: <https://www.prescqipp.info/our-resources/webkits/antimicrobial-stewardship/>.

35. UK Health Security Agency. English surveillance programme for antimicrobial utilisation and resistance (ESPAUR) Report 2022 to 2023: London: UK Health Security Agency; 2023 [Available from: <https://www.gov.uk/government/publications/english-surveillance-programme-antimicrobialutilisation-and-resistance-espaur-report>

36. Knowles R, Chandler C, O’Neill S, et al. A systematic review of national interventions and policies to optimize antibiotic use in healthcare settings in England. *Journal of Antimicrobial Chemotherapy* 2024:dkae061. doi: 10.1093/jac/dkae061

37. Bundesministerium für Soziales G, Pflege und Konsumentenschutz. Nationaler Aktionsplan zur Antibiotikaresistenz, 2021.

38. World Health Organization. Global Database for Tracking Antimicrobial Resistance (AMR) Country Self- Assessment Survey (TrACSS) - Austria 2023 [Available from: <https://amrcountryprogress.org/#/map-view>.

39. Food Chain Safety and Environment. Belgian “One Health” National Action Plan on the Fight Against Antimicrobial Resistance (AMR) 2020-2024, 2020.

40. World Health Organization. Global Database for Tracking Antimicrobial Resistance (AMR) Country Self- Assessment Survey (TrACSS) - Bulgaria 2023 [Available from: <https://amrcountryprogress.org/#/map-view>.

41. Ministarstvo zdravstva Republike Hrvatske. Nacionalni program za kontrolu otpornosti bakterija na antibiotike 2017. – 2021, 2017.

42. World Health Organization. Global Database for Tracking Antimicrobial Resistance (AMR) Country Self- Assessment Survey (TrACSS) - Croatia 2023 [Available from: <https://amrcountryprogress.org/#/map-view>.

43. Υπουργείο Υγείας. Εθνική Στρατηγική Κύπρου για την Αντιμετώπιση της Μικροβιακής Αντοχής στα Αντιβιοτικά, 2012.

44. World Health Organization. Global Database for Tracking Antimicrobial Resistance (AMR) Country Self- Assessment Survey (TrACSS) - Cyprus 2023 [Available from: <https://amrcountryprogress.org/#/map-view>.

45. World Health Organization. Global Database for Tracking Antimicrobial Resistance (AMR) Country Self- Assessment Survey (TrACSS) - Czechia 2023 [Available from: <https://amrcountryprogress.org/#/map-view>.

46. The Danish Ministry of Health. National action plan on antibiotics in human health care - Three measurable goals for a reduction of antibiotic consumption towards 2020, 2017.

47. World Health Organization. Global Database for Tracking Antimicrobial Resistance (AMR) Country Self- Assessment Survey (TrACSS) - Estonia 2023 [Available from: <https://amrcountryprogress.org/#/map-view>.

48. World Health Organization. Global Database for Tracking Antimicrobial Resistance (AMR) Country Self- Assessment Survey (TrACSS) - Finland 2023 [Available from: <https://amrcountryprogress.org/#/map-view>.

49. Ministry of Social Affairs and Health. The National Action Plan on Antimicrobial Resistance 2017–2021, 2017.

50. Ministère des Solidarités et de la Santé. 2022-2025 National Strategy for Preventing Infections and Antibiotic Resistance, 2022.

51. Federal Ministry of Health. DART 2020 - Fighting antibiotic resistance for the good of both humans and animals, 2015.

52. World Health Organization. Global Database for Tracking Antimicrobial Resistance (AMR) Country Self- Assessment Survey (TrACSS) - Germany 2023 [Available from: <https://amrcountryprogress.org/#/map-view>.

53. Προϊόντα ΚΦ. ΕΘΝΙΚΟ ΣΧΕΔΙΟ ΔΡΑΣΗΣ ΓΙΑ ΤΗΝ ΑΝΤΙΜΕΤΩΠΙΣΗ ΤΗΣ ΜΙΚΡΟΒΙΑΚΗΣ ΑΝΤΟΧΗΣ ΣΤΗΝ ΕΛΛΑΔΑ ΣΤΟ ΠΛΑΙΣΙΟ ΤΗΣ ΕΝΙΑΙΑΣ ΥΓΕΙΑΣ 2019-2023, 2019.

54. World Health Organization. Global Database for Tracking Antimicrobial Resistance (AMR) Country Self- Assessment Survey (TrACSS) - Hungary 2023 [Available from: <https://amrcountryprogress.org/#/map-view>.

55. Egészségügyi ellátórendszer szakmai módszertani fejlesztése. Infekciókontroll és AMR szakpolitikai program, az „Egy Egészség” megközelítés alapján egységben az állatgyógyászati készítményekkel, 2019.

56. World Health Organization. Global Database for Tracking Antimicrobial Resistance (AMR) Country Self- Assessment Survey (TrACSS) - Iceland 2023 [Available from: <https://amrcountryprogress.org/#/map-view>.

57. Starfshópur velferðarráðuneytis. Greinargerð starfshóps um aðgerðir til að draga úr útbreiðslu sýklalyfjaónæmra baktería á Íslandi, 2017.

58. Health Service Executive. Health Service Executive 2022-2025 AMRIC Action Plan, 2021.

59. Ministero della Salute. Piano Nazionale di Contrasto all’Antibiotico-Resistenza (PNCAR) 2022-2025, 2021.

60. Ministru kabineta. Antimikrobiālās rezistences ierobežošanas un piesardzīgas antibiotiku lietošanas plāns "Viena veselība" 2023. –2027, 2023.

61. Lietuvos Respublikos Sveikatos Apsaugos Ministras. Dėl antimikrobinėms medžiagoms atsparių mikroorganizmų plitimo prevencijos ir kontrolės 2017–2021 metų veiksmų plano patvirtinimo, 2017.

62. Santé Mdl. Plan National Antibiotiques 2018-2022 (prolongé jusqu’en 2024), 2018.

63. Ministry For Health, Ministry For Agriculture Fisheries And Animal Rights. A Strategy and Action Plan for the Prevention and Containment of Antimicrobial Resistance in Malta 2020 - 2028, 2020.

64. The Minister of Health Welfare and Sport. Netherlands: Dutch national action plan on AMR 2015-2019, 2015.

65. Norwegian Ministries. National Strategy against Antibiotic Resistance 2015–2020, 2015.

66. World Health Organization. Global Database for Tracking Antimicrobial Resistance (AMR) Country Self- Assessment Survey (TrACSS) - Poland 2023 [Available from: <https://amrcountryprogress.org/#/map-view>.

67. Ministra Zdrowia. Narodowy program ochrony antybiotyków na lata 2016-2020, 2016.

68. Ministério da Saúde, Ministério Agricultura Florestas e Desenvolvimento Rural, Ministério do Ambiente e Transição Energética. Plano nacional de combate à resistência aos antimicrobianos 2019-2023, 2019.

69. Ministerul Sănătății. Plan de acțiuni pentru implementarea Strategiei pentru prevenirea și limitarea infecților asociate asistenței medicale și combaterea fenomenului de rezistență la antimicrobiene în România 2023-2030, 2023.

70. World Health Organization. Global Database for Tracking Antimicrobial Resistance (AMR) Country Self- Assessment Survey (TrACSS) - Slovak-Republic 2023 [Available from: <https://amrcountryprogress.org/#/map-view>.

71. Minister of Health. National Action Plan on Antimicrobial Resistance in the Slovak Republic for the period 2019–2021, 2019.

72. Ministrstvo za zdravje. DRŽAVNA STRATEGIJA »ENO ZDRAVJE« ZA OBVLADOVANJE ODPORNOSTI MIKROBOV (2019-2024), 2019.

73. Agencia Española de Medicamentos y Productos Sanitarios. Plan Nacional frente a la Resistencia a los Antibióticos (PRAN) 2022-2024, 2022.

74. Ministry of Health and Social Affairs. Swedish Strategy to Combat Antibiotic Resistance 2024-2025 (extension): The government od Sweden, 2023.

75. Federal Office of Public Health and Federal Food Safety and Veterinary Office. Swiss Antibiotic Resistance Report 2022. Usage of Antibiotics and Occurrence of Antibiotic Resistance in Switzerland, 2022.

76. World Health Organization. Global Database for Tracking Antimicrobial Resistance (AMR) Country Self- Assessment Survey (TrACSS) - Switzerland 2023 [Available from: <https://amrcountryprogress.org/#/map-view>.

77. HM Government UK. Tackling antimicrobial resistance 2019–2024: The UK’s five-year national action plan, 2019.
