# Supplementary material for: Setting targets for antibiotic use in general practice in Europe: A scoping review
Source: Eur J Gen Pract. 2024 Nov 28;30(1):2430507. doi: 10.1080/13814788.2024.2430507 (PMC11610282; doi:10.1080/13814788.2024.2430507)
Supplement: Supplemental Material [file IGEN_A_2430507_SM3297.zip › ejgp-2024-0174-File008.docx]

Supplementary material 6. National targets without local targets (general practices)

| **Country** | **National/regional targets** |
| --- | --- |
| Belgium | **Outpatient practice/Ambulatory care**  *2014 to 2019*  - Reduce the consumption of quinolones from 10% of total antibiotic consumption to 5% by 2018 [1-4]  - Increase in the ratio of amoxicillin versus amoxicillin-clavulanic acid to 80/20 by 2018 [1-4]  *2020 to 2024*  - Reduce the total consumption of antibiotics prescriptions per 1,000 inhabitants from 800 to 400 by 2025 [1-4]  - Reduce 40% of antibiotic consumption [1]  - Reduce the consumption of quinolones to a maximum of 5% of total antibiotic consumption by 2024 [1]  - Increase the ratio of amoxycillin to amoxycillin/clavulanic acid to 80/20 [1] |
| Denmark | **Primary care**  2016 to 2020:  - Reduce antibiotic prescriptions per 1000 inhabitants from 460 to 350 [5,6]  - Increase the use of Penicillin V from 31% to 36 % of the total antibiotic prescriptions per 1000 inhabitants [5,6] |
| Greece | 2019 to 2023  **Community care**  - Reduce the consumption of antibiotics by 25%. The aim is for the consumption to fall below the value of 30 defined daily doses (DDD)/1000 inhabitants [7]  **Out-hospital environment**  - Maximum 30 DDD/1000 population of quinolones for respiratory infections [7] |
| Italy | Local area [8]  - Reduction of ≥10% in consumption (DDD/1000 inhab. day) of systemic antibiotics in 2025 compared to 2022  - Reduction of ≥20% in the ratio between consumption (DDD/1000 inhab day) of broad-spectrum molecules and narrow-spectrum molecules in 2025 compared to 2022.  *Population pediatric* [8]  - Increase ≥30% ratio amoxicillin/amoxicillin+clavulanic acid prescriptions  - Reduction of ≥10% in consumption (DDD/1000 inhab. day) of systemic antibiotics in the local area in 2025 compared to 2022  - Reduction of ≥20% in the ratio between consumption (DDD/1000 inhab day) of broadspectrum molecules and narrow-spectrum molecules in 2025 compared to 2022. |
| Netherlands | 2015 to 2019:  Maximum 50% of the use of incorrectly prescribed antibiotics across the entire healthcare chain, relative to a baseline determined with stakeholders [3,9] |
| Norway | *2015 to 2022*  **Ambulatory care**  - Reduce antibiotic consumption by 30% by 2020 compared with 2012 consumption [3,4,10]  - Reduce antibiotic prescriptions per 1000 inhabitants from 450 to 250 by 2020 [3,4,10]  - Reduce antibiotic prescriptions to treat respiratory infections by 20% by 2020 compared with 2012 [3,4,10]  Target from the national treatment *guidelines and the National Antibiotics Committee*  - Increase of phenoxymethylpenicillin prescribed for respiratory tract infections in children aged 0–6 years to 80% of all antibiotics prescribed for respiratory tract infections in the same patient group [3]  - Reduce fluoroquinolones (and, in particular, ciprofloxacin) for treating uncomplicated urinary tract infections in women aged 20–79 years to less than 8% of all antibiotics prescribed for urinary tract infections in the same patient group [3]  - A 30% reduction of antibiotic prescriptions for respiratory tract infections in children aged 0–6 years [3] |
| Portugal | Goals to be achieved by 2023: Antibiotic consumption in the community = 17.00 DDD [11] |
| Slovenia | *2019 to 2024*  **Ambulatory care**  - Reduce the total ambulatory use of antibiotics by 20% compared to 2017 [3,12]  - Reduce ambulatory use of broad-spectrum antibiotics such as amoxicillin with clavulanic acid, fluoroquinolones, macrolides with a long half-life (azithromycin), and 2nd and 3rd generation cephalosporins [3,12] |

**References**

[1] Food Chain Safety and Environment. Belgian “One Health” National Action Plan on the Fight Against Antimicrobial Resistance (AMR) 2020-2024. 2020.

[2] Bruyndonckx R, Coenen S, Hens N, et al. Antibiotic use and resistance in Belgium: the impact of two decades of multi-faceted campaigning. Acta Clin Belg. 2021 Aug;76(4):280-288.

[3] D'Atri F, Arthur J, Blix HS, et al. Targets for the reduction of antibiotic use in humans in the Transatlantic Taskforce on Antimicrobial Resistance (TATFAR) partner countries. Euro Surveill. 2019 Jul;24(28).

[4] Howard P, Huttner B, Beovic B, et al. ESGAP inventory of target indicators assessing antibiotic prescriptions: a cross-sectional survey. J Antimicrob Chemother. 2017 Oct 1;72(10):2910-2914.

[5] The Danish Ministry of Health. National action plan on antibiotics in human health care - Three measurable goals for a reduction of antibiotic consumption towards 2020. 2017.

[6] Michalsen BO, Xu AXT, Alderson SL, et al. Regional and national antimicrobial stewardship activities: a survey from the Joint Programming Initiative on Antimicrobial Resistance—Primary Care Antibiotic Audit and Feedback Network (JPIAMR-PAAN). JAC-Antimicrobial Resistance. 2023;5(2).

[7] Προϊόντα ΚΦ. ΕΘΝΙΚΟ ΣΧΕΔΙΟ ΔΡΑΣΗΣ ΓΙΑ ΤΗΝ ΑΝΤΙΜΕΤΩΠΙΣΗ ΤΗΣ ΜΙΚΡΟΒΙΑΚΗΣ ΑΝΤΟΧΗΣ ΣΤΗΝ ΕΛΛΑΔΑ ΣΤΟ ΠΛΑΙΣΙΟ ΤΗΣ ΕΝΙΑΙΑΣ ΥΓΕΙΑΣ 2019-2023. 2019.

[8] Ministero della Salute. Piano Nazionale di Contrasto all’Antibiotico-Resistenza (PNCAR) 2022-2025. 2021.

[9] The Minister of Health Welfare and Sport. Netherlands: Dutch national action plan on AMR 2015-2019. 2015.

[10] Norwegian Ministries. National Strategy against Antibiotic Resistance 2015–2020. 2015.

[11] Ministério da Saúde, Ministério Agricultura Florestas e Desenvolvimento Rural, Ministério do Ambiente e Transição Energética. Plano nacional de combate à resistência aos antimicrobianos 2019-2023. 2019.

[12] Ministrstvo za zdravje. DRŽAVNA STRATEGIJA »ENO ZDRAVJE« ZA OBVLADOVANJE ODPORNOSTI MIKROBOV (2019-2024). 2019.
