# Supplementary material for: Setting targets for antibiotic use in general practice in Europe: A scoping review
Source: Eur J Gen Pract. 2024 Nov 28;30(1):2430507. doi: 10.1080/13814788.2024.2430507 (PMC11610282; doi:10.1080/13814788.2024.2430507)
Supplement: Supplemental Material [file IGEN_A_2430507_SM3297.zip › ejgp-2024-0174-File009.docx]

# Supplementary material 7. SMART (specific, measurable, accurate, realistic and time-bound) criteria and target-setting checklist

| **ID record** | **Targets** | **Setting** | **SMART criteria** | | | | | **Target-setting Checklist** | | | | | | | | |
| --- | --- | --- | --- | --- | --- | --- | --- | --- | --- | --- | --- | --- | --- | --- | --- | --- |
|  |  |  | **Specific** | **Measurable** | **Accurate** | **Realistic** | **Time bound** | **What was the starting position?** | **What was the trend?** | **How did trends compare with other areas?** | **How might target be affected by other local or national initiatives?** | **Had staff providing services been consulted on the target, and are they committed to its achievement?** | **Was the target worthwhile?** | **Was there a plan to collect, analyse, and verify the data?** | **Data collection cost** | **Compared to achievements elsewhere, did reaching the target represent value for money?** |
| D'Atri 2019 [1]  Howard 2017 [2]  Bruyndonckx 2021 [3]  NAP [4] | - Reduce the total consumption of antibiotics prescriptions per 1,000 inhabitants from 800 to 400 by 2025  - Reduce 40% of antibiotic consumption (1)  - Reduce the consumption of quinolones to a maximum of 5% of total antibiotic consumption by 2024  - Increase the ratio of amoxycillin to amoxycillin/clavulanic acid to 80/20 | Belgium: Outpatient practice/Ambulatory care | Specific for antibiotic group | These targets were based on historical indicators already being monitored | Based on data | Based on data | They had a target each year and modified it | They provided a reference value and their progress and achievement | These targets were based on historical indicators. They have informed their progress and achievement | Not provided information to check this item | They had a national cost-effective web-based communication skills training to support general practitioners to safely reduce antibiotic prescribing. | Not provided information to check these items | | | | |
| NAP [5] | - Reduce the total consumption of all antibiotics by at least 20 defined daily doses (DDD) per 1,000 inhabitants per day by 2025  - Maximum 20% of antibiotic treatments per 100 patients (attending physician) aged 16 to 65 years old and with no long-term illness  - Reduce to 650 or less the number of antibiotic prescriptions dispensed in community care (per 1,000 inhabitants and per year)  - Maximum 20% of the consumption of all critical antibiotics for systemic use in community care, in DDD per 1,000 inhabitants per day, dispensed in community pharmacies by 2025 | France: Community care | Specific for antibiotic and age group | National Public Health Agency collected data and monitored these targets | Based on existing surveillance data and some were set arbitrarily on the basis of expert advice in relation to expected outcomes | Based on existing surveillance data and some were set arbitrarily on the basis of expert advice in relation to expected outcomes | They specified a time for each a target | 2019 value was the reference value (baseline) | They specified the change in indicator from 2015- 2019 | Not provided information to check this item | Incentive to encourage healthcare professionals to improve prescription practices | Indicator dashboard with targets to guide the national/regional/local strategy | Not provided information to check these items | | | |
| D'Atri 2019 [1]  Howard 2017 [2] | - Reduce to 14 treatments per 100 patients the annual antibiotic prescription rate in patients aged 16–65 years without a chronic disease  - Maximum 27% of patients treated with ‘critical antibiotics’ (amoxicillin-clavulanic acid, third- and fourth generation cephalosporins, fluoroquinolones)  - Maximum 3% of children treated with third generation cephalosporins in one year, out of children receiving antibiotics (any child aged < 4 years)  - Maximum 2% of children treated with third generation cephalosporins in one year, out of children receiving antibiotics (any child aged ≥4 years) | Frace: primary care | Specific for antibiotic and age group | The French Medicines Agency (ANSM) compiled data on overall antibiotic consumption | Unclear | Unclear | 2011 to 2017 | Not provided information to check these items | | | | | | | | |
| NAP [6] | **Community care**  Reduce antibiotic prescribing by 2% each year to achieve a target of 20.5 DDD by 2025  **General practices**  - Reduce “red” antibiotics by 8% (to 26%) by 2025  - Reduce the total number of antibiotic prescriptions by 8% for patients receiving free healthcare by 2025 | Ireland: Community care/ General practices | Specific for antibiotic group | Unclear | Based on GP data  (issue 10 GP reports) | Based on GP data  (issue 10 GP reports) | Each year until 2025 | - For 2022, community consumption of antibiotics of <22 DDDs per 1,000 population  - By the end of 2021 Q1 34% (previous 12 months data) of antibiotics prescribed in General Practice | Not provided information to check these items | | | | | | | |
| Swedish NAP [7]  Swedres-Svarm 2020 [8]  D'Atri 2019 [1]  Public Health Agency of Sweden 2014 [9]  Public Health Agency of Sweden 2019 [10]  Molstad 2017 [11] | - Maximum 250 antibiotic prescriptions issued per 1,000 inhabitants per year  - Minimum of 80% of all antibiotics used to treat respiratory tract infections in children aged 0–6 years should be penicillin V (J01CE02)  - Maximum 10% of all antibiotics used to treat urinary tract infections in women aged 18–79 years should be fluoroquinolones  - Maximum 10% of patients with acute bronchitis should receive antibiotic treatment  - Maximum 80% of women and more than 50% of men with afebrile urinary tract infection should receive first-line treatment  - Maximum 90% of patients treated against pharyngotonsillitis should receive penicillin V | Sweden: Primary care/Outpatient care/Ambulatory care | Specific for age, sex and disease groups | Antibiotic sales data were collected and sent by the Public Health Agency of Sweden to the Strama Programme Council and to each local Strama group. The targets are also monitored via the Primary Care Quality national register. | Targets were based on evidence-based guidelines and supported by data | Targets were based on evidence-based guidelines and supported by data | Unclear, the time taken to achieve the target was not specified | Unclear, a reference value or baseline | Unclear if it was the trend however, calculations based on current Swedish epidemiological data | Primary Care Quality system: Benchmarks can be shown for each indicator on local, regional and national levels | They had local initiatives such as quality system and financial incentives | They provided local incentives | Not provided information | Yes | Not provided information to check these items | |
| Public Health Agency of Sweden 2014 [9] | - Maximum 20% of all acute bronchitis should be treated with antibiotics  - Over 70% of all patients treated with antibiotics for pneumonia should receive penicillin V  - Maximum 3% of women over 18 years of age who are treated with quinolones when diagnosed with cystitis. The same applies to the proportion of women with cystitis who are treated with cephalosporins | Sweden: General practices | Specific for age, sex and disease groups | These were registered and monitored by Primary Care Quality tool | Targets were based on evidence-based guidelines and supported by data | Targets were based on evidence-based guidelines and supported by data | Unclear, the time taken to achieve the target was not specified | Unclear, a reference value or baseline | Unclear if it was the trend however, calculations based on diagnosis-linked data | Primary Care Quality system: Benchmarks can be shown for each indicator on local, regional and national levels | They had local initiatives such as quality system and financial incentives | They provided local incentives | Not provided information | Yes | Not provided information to check these items | |
| Nathwani 2011 [12]  Nathwani 2012 [13] | Seasonal variation in quinolone use (summer months (April–September) vs. winter months (October–March)) is < 5% | UK - Scotland: primary care and general practice | Specific for Seasonal variation and Fluoroquinolones. | NHS Boards formulary should be subject to regular review and monitoring and exception prescribing should be monitored | Target was based on evidence-based guidelines and supported by data on antibiotic prescribing primary care. | It did not give a timeframe to achievable | Unclear | The baseline was the annual seasonal variation in the use of fluoroquinolones from 2005 to 2008 | Trend from 2005 to 2008 | Trends compared between all NHS boards | These national targets in turn led to local primary care-based interventions within Health Boards | Local Antimicrobial Management Teams are subgroups | Not provided information | NHS Boards formulary should be subject to regular review and monitoring and exception prescribing should be monitored | Not provided information to check these items | |
| UK Health Security Agency 2023 [14]  Niazi-Ali 2022 [15]  Howard 2017 [2]  Gold 2022 [16]  D'Atri 2019 [1] | - Maximum 1.161 items per STAR-PU of the 2013-2014 baseline mean performance value for England (a 1% reduction). Reduced to 0.965 in 2019-2020 (36, 40). In 2022-2023, it was further reduced to 0.871 items per STAR-PU  - Maximum 10% of broad-spectrum antibiotic prescribing (co-amoxiclav, cephalosporin class and fluoroquinolone)  - Reduce inappropriate antibiotic prescribing for urinary tract infections, the target for 2017–18 (based on June 2015–May 2016 baseline data) includes a minimum 10% reduction in the trimethoprim/nitrofurantoin prescribing ratio and a 10% decrease in trimethoprim prescriptions for patients aged ≥ 70 years due to higher trimethoprim non-susceptibility rates in this age group | UK - England: primary care and general practice | Specific for disease, antibiotic group and age | Data was collected for each GP practice on a monthly basis by the NHS Business Services Authority. Public Health England makes these data available on Fingertips | Target was based on data on antibiotic prescribing primary care. | Target was based on data on antibiotic prescribing primary care. | They had a target each year and modified it | Baseline data of the previous year | Fingertips is able to monitor the trend of an indicator over time | Fingertips allow local users to benchmark their data against the national and/or regional picture or against comparable organizations | They had local initiatives such as fingertips and financial incentives. The provision of data relating to AMR local indicators via Fingertips is a valuable tool for facilitating development, implementation and monitoring of local action plans | They provided local finance incentives and Quality Premium improvement measures | They showed a reduction of antibiotics prescription primary care and have adjusted of these targets over time | The antibiotic prescribing trends are monitored monthly. Fingertips is able to monitor the trend of an indicator over time | Not provided information to check these items | |
| D'Atri 2019 [1] | Practices must achieve an equivalent or lower prescribing rate to the Scottish 25th percentile or reduce their prescribing rate by at least one-fifth of the national interquartile range | UK - Scotland: primary care | Not specific | Scottish Antimicrobial Resistance and Healthcare Associated Infection (SARHAI) group monitored this target | Based on data | Based on data | Unclear | Not specified a reference value or baseline | Not specified the trend | Not trends comparison | Not provided information to check these items | | | | | |
| British Society for Antimicrobial Chemotherapy 2018 [17] | The report provided to Scottish general practices compares their prescribing data to benchmarks based on the 25th percentile, representing the antibiotic prescribing rate achieved or surpassed by the lowest-prescribing quarter of practices within their local National Health Service board and across Scotland as a whole (40) | UK - Scotland: general practice | Not specific | Local NHS board monitored this target | Unclear | Unclear | Unclear | Not specified a reference value or baseline | Not specified the trend | Not trends comparison | Not provided information to check these items | | | | | |

References

[1] D'Atri F, Arthur J, Blix HS, et al. Targets for the reduction of antibiotic use in humans in the Transatlantic Taskforce on Antimicrobial Resistance (TATFAR) partner countries. Euro Surveill. 2019 Jul;24(28).

[2] Howard P, Huttner B, Beovic B, et al. ESGAP inventory of target indicators assessing antibiotic prescriptions: a cross-sectional survey. J Antimicrob Chemother. 2017 Oct 1;72(10):2910-2914.

[3] Bruyndonckx R, Coenen S, Hens N, et al. Antibiotic use and resistance in Belgium: the impact of two decades of multi-faceted campaigning. Acta Clin Belg. 2021 Aug;76(4):280-288.

[4] Food Chain Safety and Environment. Belgian “One Health” National Action Plan on the Fight Against Antimicrobial Resistance (AMR) 2020-2024. 2020.

[5] Ministère des Solidarités et de la Santé. 2022-2025 National Strategy for Preventing Infections and Antibiotic Resistance. 2022.

[6] Health Service Executive. Health Service Executive 2022-2025 AMRIC Action Plan. 2021.

[7] Ministry of Health and Social Affairs. Swedish Strategy to Combat Antibiotic Resistance 2024-2025 (extension). The government od Sweden; 2023.

[8] Swedres-Svarm. Sales of antibiotics and occurrence of antibiotic resistance in Sweden. Solna/Uppsala; 2020.

[9] Public Health Agency of Sweden. Swedish work against antibiotic resistance Tools, working methods and experiences 2014. Available from: <https://strama.se/strategiska-dokument/>

[10] Public Health Agency of Sweden, STRAMA. Strama's goals for antibiotic use in outpatient care 2019. Available from: <https://strama.se/strategiska-dokument/>

[11] Molstad S, Lofmark S, Carlin K, et al. Lessons learnt during 20 years of the Swedish strategic programme against antibiotic resistance. Bull World Health Organ. 2017 Nov 1;95(11):764-773.

[12] Nathwani D, Sneddon J, Malcolm W, et al. Scottish Antimicrobial Prescribing Group (SAPG): development and impact of the Scottish National Antimicrobial Stewardship Programme. Int J Antimicrob Agents. 2011 Jul;38(1):16-26.

[13] Nathwani D, Sneddon J, Patton A, Malcolm W. Antimicrobial stewardship in Scotland: impact of a national programme. Antimicrob Resist Infect Control. 2012 Feb 3;1(1):7.

[14] UK Health Security Agency. English surveillance programme for antimicrobial utilisation and resistance (ESPAUR) Report 2022 to 2023: London: UK Health Security Agency; 2023. Available from: <https://www.gov.uk/government/publications/english-surveillance-programme-antimicrobialutilisation-and-resistance-espaur-report>

[15] Niazi-Ali S, Bircher J. Broad spectrum antibiotic stewardship by quality improvement methods. Int J Risk Saf Med. 2022;33(S1):S35-S40.

[16] Gold N, Sallis A, Saei A, et al. Using text and charts to provide social norm feedback to general practices with high overall and high broad-spectrum antibiotic prescribing: a series of national randomised controlled trials. Trials. 2022 Jun 18;23(1):511.

[17] British Society for Antimicrobial Chemotherapy. Antimicrobial Stewardship: From Principles to Practice. 2018.
